# Aerial and underwater drones for marine litter monitoring in shallow coastal waters: factors influencing item detection and cost-efficiency

**DOI:** 10.1007/s10661-022-10519-5

**Published:** 2022-10-11

**Authors:** Gabriela Escobar-Sánchez, Greta Markfort, Mareike Berghald, Lukas Ritzenhofen, Gerald Schernewski

**Affiliations:** 1 Coastal Research and Management Group, Leibniz Institute for Baltic Sea Research, Seestraße 15, 18119 Warnemünde, Germany; 2 Marine Research Institute of Klaipeda University, Universiteto ave. 17, 92294 Klaipeda, Lithuania

**Keywords:** UAV, ROV, Marine litter, Marine Strategy Framework Directive, Monitoring, Cost-efficiency

## Abstract

Although marine litter monitoring has increased over the years, the pollution of coastal waters is still understudied and there is a need for spatial and temporal data. Aerial (UAV) and underwater (ROV) drones have demonstrated their potential as monitoring tools at coastal sites; however, suitable conditions for use and cost-efficiency of the methods still need attention. This study tested UAVs and ROVs for the monitoring of floating, submerged, and seafloor items using artificial plastic plates and assessed the influence of water conditions (water transparency, color, depth, bottom substrate), item characteristics (color and size), and method settings (flight/dive height) on detection accuracy. A cost-efficiency analysis suggests that both UAV and ROV methods lie within the same cost and efficiency category as current on-boat observation and scuba diving methods and shall be considered for further testing in real scenarios for official marine litter monitoring methods.

**Supplementary Information:**

The online version contains supplementary material available at 10.1007/s10661-022-10519-5.

## Introduction

Coastal waters, defined as sea waters reaching up to one nautical mile from land (WFD 2000/60/EC) and 20 m depth (Schernewski et al., 2004), are subject to a variety of anthropogenic pressures including marine litter pollution. Marine litter (ML) is defined as any persistent solid material that enters the marine and coastal environment deliberately or accidentally from land or sea based sources (UNEP, 2005), such as fishing, shipping, tourism, and coastal recreation (Galgani et al., 2015). Latest estimations suggest that annually 5.1 million tons of mismanaged plastic enter the oceans globally from direct coastal inputs and the transport of rivers (which represent 1.4 million tons per year alone) (Chassignet et al., 2021).

ML accumulates in three main physical compartments: beaches, seawater, and seafloor (GESAMP, 2019). The force of wind, waves, and currents cause the transport and further accumulation between these compartments (Browne et al., 2010; van Sebille et al., 2020). Surface area, size, and material density of the items as well as bottom roughness and bathymetry of the site will influence their transport, behavior, and deposition (Browne et al., 2010; Schwarz et al., 2019; van Sebille et al., 2020). In waterbodies, this leads to mainly two types of litter: floating and sinking litter. Floating litter, composed of buoyant items (density < 1.03 g/cm^3^ of seawater), is transported by wind, waves, and currents and often ending at the shore, river banks, or trapped in coastal vegetation such as reed belts. In contrast, sinking litter, composed of non-buoyant items (density > 1.03 g/cm^3^ of seawater), will accumulate on the seafloor and be permanently trapped in sediments and underwater habitats or structures (Galgani et al., 2015; van Sebille et al., 2020). It is estimated that 75% of litter inputs in the oceans is beached, while 25% remain in seawater (Chassignet et al., 2021). At coastal waters, it is likely that this percentage of floating litter is less due to presence of stronger currents, wave movement, and higher phytoplankton activity than offshore, leading to stronger vertical mixing and biofouling causing floating litter to either beach or rapidly sink (van Sebille et al., 2020). In this sense, coastal surface waters mainly serve as temporary sinks and transition areas of ML, while the seafloor is likely a permanent sink (Koelmans et al., 2017; Lebreton et al., 2017; van Sebille et al., 2020).

Enclosed seas such as the Mediterranean and the Baltic are hotspots of ML due to a high population density, high number of coastal and marine activities, and low water exchange (Chassignet et al., 2021; Kaandorp et al., 2020; Schernewski et al., 2017, 2021). In the Mediterranean Sea, litter inputs come mainly from the coastal population, rivers, and fisheries (Kaandorp et al., 2020), while in the Baltic Sea, tourism and recreation are the predominant sources of macrolitter (Schernewski et al., 2017). Plastic transport models for the southern Baltic Sea show that macrolitter from touristic beaches accumulates back at the shore within days (Schernewski et al., 2017), whereas in the Mediterranean, the highest beaching of plastic occurs at coasts of North Africa and sinking of litter majorly occurs next to the coast of European countries (Kaandorp et al., 2020).

The European Marine Strategy Framework Directive (MSFD 2008/56/EC) provides a regulatory legislation for the protection of the marine environment through the assessment and monitoring of the environmental status of marine waters, identifying the necessary measures to achieve or maintain a good environmental status (GES) in all European seas. A GES is evaluated based on 11 descriptors. Descriptor 10 refers to ML and the identification of trend amounts, accumulation sites, composition, spatial distribution, and sources of litter pollution at the coastline, water column, and seafloor, as well as impacts in biota (JRC, 2011). Based on the latest report of the EU Commission on the implementation of the MSFD, there are still major research gaps on litter quantities, pathways and sources in surface waters, water column, and seafloor (EC, 2020). These research gaps are present in both the Mediterranean and the Baltic Sea. In the Mediterranean Sea, lack of monitoring data and management of litter in North-African countries affects the progress to achieve a GES. Similarly, in the Baltic Sea, more temporal and spatial data is required to obtain better modeling estimations and understand emission, transport, deposition, and retention of plastics (Schernewski et al., 2017, 2021).

Monitoring methods shall be widely applicable, comparable, transferable, and cost-efficient, to derive solutions and evaluate their effectiveness. There is a need of large spatial and temporal data coverage and further development of automatic methods for image and video analysis (JRC, 2013; EC, 2020), for example, with aerial (UAV) and underwater (ROV) drones. Consequently, the urgent time frame to achieve a consistent ML monitoring in Europe require an assessment of the applicability and cost-efficiency of drone technologies as alternative methods.

### Potential of UAVs and ROVs for marine litter monitoring

Current monitoring methods for floating litter include on-ship surveys for sizes between 2.5 and 50 cm, riverine monitoring, e.g., from bridges, and aerial surveys with airplanes which are rather applicable to the higher litter size range > 30 cm (JRC, 2013). For seafloor litter in shallow waters, scuba diving or snorkeling are common survey methods (JRC, 2013). Although these methods are widely applicable and validated, they can be time intensive, require high costs, expertise, and collaboration to combine efforts. Researchers highlight the need to use standardized methodologies for monitoring (e.g., items per area) that enable comparison with other studies (Spengler & Costa, 2008; Topouzelis et al., 2021)—here remote-sensing methods come into question.

Aerial remote sensing of floating ML with satellites and airplanes has a longer trajectory than with aerial drones (UAVs). Examples are the use of RGB (visible light spectrum) cameras on aircrafts for the detection of ghost drifting nets (Pichel et al., 2012), RGB and hyperspectral (SWIR) sensors for the detection of floating plastics in the Great Pacific Garbage Patch (Garaba et al., 2018), and multispectral satellite data for the detection of exemplary plastic patches of PET bottles and LDPE bags (Topouzelis et al., 2020). These methods, however, detect large litter sizes of 10–25 m (e.g., ship wrecks, ghost nets, and litter patches) and due to lower resolution, do not serve to derive item types, materials, and sources of pollution. Other limitations are the higher costs of implementation and overall higher complexity of image analysis (Topouzelis et al., 2021).

UAVs has been successfully used for detecting floating macrolitter (> 2.5 cm) using different cameras and image analysis techniques. Geraeds et al. (2019) compared UAVs to visual survey and manta trawl to estimate floating macrolitter (> 2.5 cm) abundance in a river in Malaysia at altitudes of 5–15 m, involving citizen science for manual image screening. They observed a similar pattern in spatiotemporal distribution of litter with both methods; however, the number of items observed with UAV surveys was higher, likely since the manual screening allowed more time for observation. Similarly, Atwood et al. (2018) used an UAV at flight heights of 30–60 m to map floating litter patches in a river in Indonesia, using machine learning to classify litter from vegetation, sun glint, and water reaching an accuracy of 68–90%. Studies in the Mediterranean Sea, tested UAVs at altitudes of 65 m to detect floating macrolitter (> 20 cm) in comparison to on-ship observation, suggesting similar macrolitter densities obtained with both methods. Main limitation for the UAV was the categorization of items (Garcia-Garin et al., 2020a). Most recently, Garcia-Garin et al. (2021) tested convolutional neural networks (CNN) to detect floating litter at sea from RGB images obtained with an UAV and aircraft at altitudes of 20–300 m, obtaining a detection accuracy > 81%. Hyperspectral and thermal infrared sensors on UAVs have also been recently tested. Freitas et al. (2021) used an hyperspectral sensor on aircraft and an UAV to detect plastic patches (10 × 10 m) of LDPE, white plastic film, and rope, achieving similar results in spectral signatures between vehicles. Another study was able to detect floating plastic (0.5 × 0.5 m) with a thermal infrared camera at 30 m altitude. The sensor can be applied at day and night and complement RGB imagery (Goddijn-Murphy et al., 2022).

To the best of our knowledge, there is currently no study testing the detection of submerged ML at shallow waters with UAVs, which is limited by water depth and transparency. The previous mentioned studies (Freitas et al., 2021; Goddijn-Murphy et al., 2022; Topouzelis et al., 2020) found a specific signature for plastic at approximately 1215 and 1732 nm; however, when the item is wet, the signal becomes weak. It is likely that for submerged items, the application of such sensors does not provide valuable data; thus, the reliance on RGB data is still important.

ROVs have been largely used for underwater ML monitoring, since these enable investigations in areas where other types of monitoring (e.g., scuba diving, snorkeling, or the use of big boats for bottom trawling) are not possible (Ioakeimidis et al., 2015; Madricardo et al., 2020). However, there is fewer studies focusing on shallow waters and small litter sizes. Ioakeimidis et al. (2015) used ROV videos to estimate abundance and types of ML in the coastal waters of the eastern Mediterranean Sea at depths of 96–113 m and classified ML into size classes (> 5 cm) and six categories: plastic, metal, rubber, ceramic/glass, natural, and miscellaneous. Similar works from Consoli et al. (2018) and Enrichetti et al. (2020) were able to estimate densities and sources of ML through manual screening of videos in the central Mediterranean Sea at depths of 5–30 m and 30–220 m, respectively, classifying ML into size classes (< 1 to > 10m^2^) and two categories: general urban waste and fishing gear. Regarding underwater image analyses, Valdenegro-Toro (2017) was one of the first to test deep learning (CNN) for detection of underwater ML using sonar data, being able to classify items into six categories: metal, glass, cardboard, rubber, plastic, and background with an accuracy of 71%. The study by Fulton et al. (2018) is perhaps one of the first to apply deep learning for the detection of ML underwater in real scenarios. The authors tested CNN using real RGB images and videos obtained from expeditions at different sites, water transparencies, years, and litter sizes and types. Classification was done in three categories: plastic, ROV parts, and biological material with promising results for real-time detection. A follow-up study tested CNN algorithms for the same purpose, reaching 88–95% accuracy. However, a common limitation mentioned in these studies is the lack of quality images for training (Moorton et al., 2022).

The environmental requirements and limitations as well as the niches for application of UAVs and ROVs as monitoring tools have so far been little discussed. There is a need to understand the sampling requirements of ML in seawater and define a standard methodology for monitoring (Martínez-Vicente et al., 2019). This study aims at testing the potential of commercial UAV and ROV drones for detecting floating, submerged, and underwater items in comparison to current methods, by (a) assessing the influence of water conditions (water depth, water transparency and bottom substrate), item characteristics (color and size) and method settings (flight height and dive depth) in item detection, (b) set thresholds for item detection, and (c) evaluate the cost-efficiency of these methods for official monitoring strategies.

## Materials and methods

### Study site

**Fig. 1:**
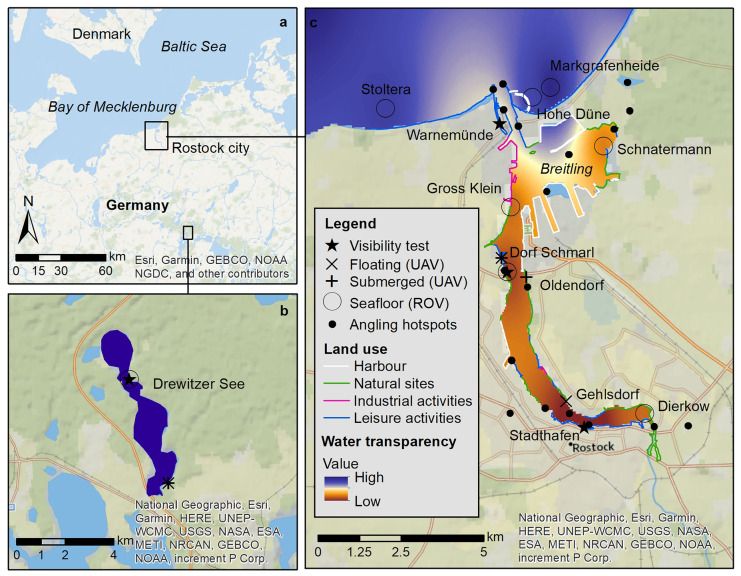
Study area: sampling sites, water transparency and land use types that could be potential marine litter inputs. Recovery experiments for floating (X), submerged (+) and underwater (O) items were carried out at coastal waters of the Baltic Sea outside of Rostock city (**a**), the Warnow estuary (**b**) and the lake Drewitzer See (**c**) in the state of Mecklenburg-Vorpommen, Germany

The experiments were carried out in Southern Baltic coastal waters. The sites had different water color, transparency, and bottom substrate to exemplify a gradient of conditions and were located in inner waters of the Warnow estuary and coastal waters near the city of Rostock in Germany. Additionally, the Drewitzer See, with high water transparency was included (Fig. 1, Table 1).

**Table 1:**
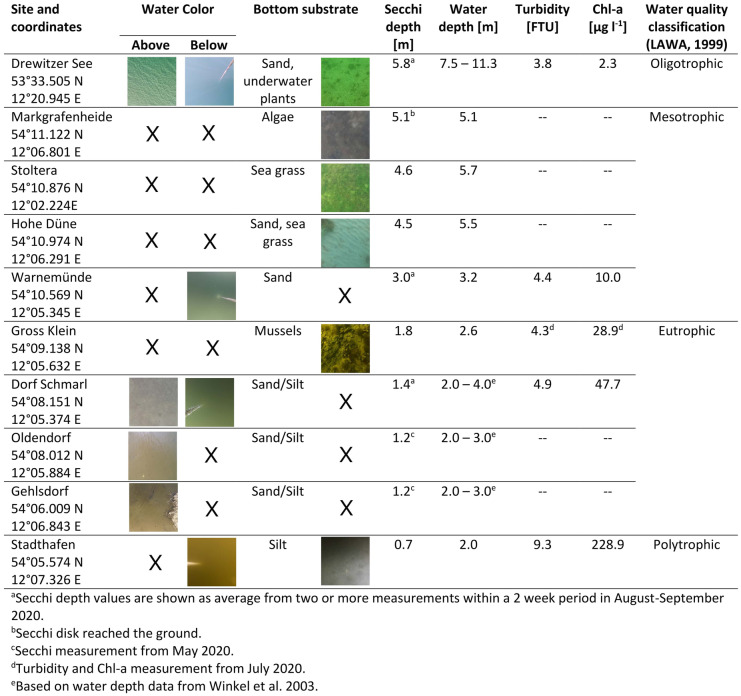
Study site characteriscs: water depth z_water_, water color, Secchi depth z_SD_, and sediment substrate. The sites were classified by water transparency, based on Secchi depth z_SD_ measurements following the water quality classification by LAWA (1999). Secchi depth was taken as the last visible depth

The Warnow estuary has an area of 12 km^2^ with a mean depth of 5.6 m (Lange et al., 2020) and a catchment area of 3,280 km^2^. It is a micro-tidal estuary, with strong varying salinity (5 PSU at river entrance up to 18 PSU at the estuary opening) (Lange et al., 2020). Inflow of river water is lower than the inflow of water from the Baltic Sea, estimated at 440 km^3^ freshwater versus 1,180 km^3^ seawater inflow per year (Szymczycha et al., 2019), having a water residence time of 30 days (Lange et al., 2020). Nutrient inputs are highly dependent on runoff from agriculture, wastewater treatment plants and rivers, with relatively high nitrogen and phosphorus inputs(HELCOM, 2018a, b; Nausch et al., 2011). Annual contents for total nitrogen, phosphorus and chloropyll-a in the upper water layer (0–10 m) were higher in the Warnow estuary than the threshold values, whereas the values for the same components in coastal waters between Warnemünde and Darß were closer to the thresholds set.^1^ Although nutrient inputs have strongly decreased since the 1990s, both coastal and inner waters are still considered under eutrophication having a moderate to bad environmental status (Nausch et al., 2011). Due to the high nutrient and sediment input along the Warnow estuary, there is a gradient of water transparency (i.e., light penetration into the water), with low transparency at the river entrance (Stadthafen, *z*_SD_ = 0.75 m) to high transparency at the estuary opening (Warnemünde, *z*_SD_ > 3.2 m). Likewise, water transparency was higher at all sites outside the estuary (*z*_SD_ = 4.5 – 5.1 m) (Fig. 1, Table 1).

Bottom substrate in the Warnow estuary is composed of sand, silt, and rock clusters. At the estuary opening at Warnemünde, bottom substrate consists mainly of fine sand, areas with rocks and stones (LUNG, 2007), as well as sea grass meadows (EOS, 2020). At coastal sites between Warnemünde and Markgrafenheide, a natural formation of sand reef appears nearshore. Here, sea grass meadows, algae, and mussel beds were also present (observed with ROV, Table 1).

The Drewitzer See lake covers an area of 6.9 km^2^, has a maximum water depth of 31 m and a water residence time of 27 years (Fig. 1) (LUNG, 2016). It is oligotrophic with high water transparency (Secchi depth, z_SD_) during the summer months of *z*_SD_ = 5.8 m (LUNG, 2006). The bottom is covered by sand and underwater plants (Table 1).

### Camera tests on visibility of items underwater

**Fig. 2:**
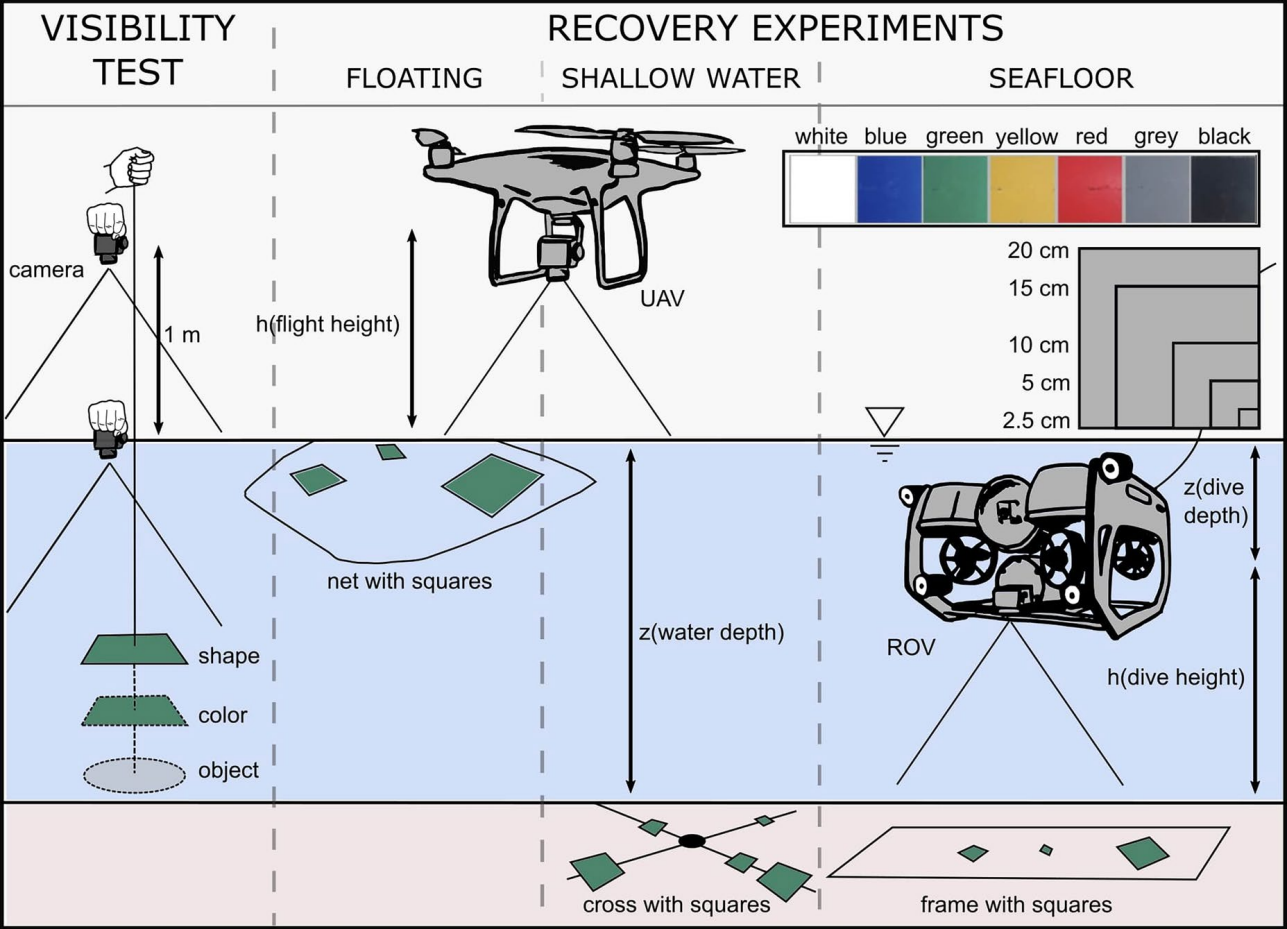
Methodology for visibility test and recovery experiments for floating, submerged and underwater items. Visibility tests aimed at evaluating the maximum visible depth at the items of 7 different colors and 3 sizes (5, 10 and 20 cm) were clearly visible. Influence in color and shape detection were also considered. Recovery experiments were tested to assess the potential of detection of floating, submerged and underwater items using 7 colors and 4 sizes (2.5 – 15 cm)

The visibility of items underwater of different color and size was measured with a bare eye test, similar to the Secchi method, to assess the influence of water conditions (transparency, color, background substrate) in item detection. PVC plastic squares of seven different colors: white, black, grey, blue, green, yellow, and red, and 3 sizes: 20 cm, 10 cm, and 5 cm were used (Fig. 2). The tests used a camera above and below the water surface and were carried out at four sites of different water transparency, namely Stadthafen, Dorf Schmarl, Warnemünde, and Drewitzer See (Fig. 1, Table 1). Secchi depth, as indicator for water transparency, was measured at each site, with a white secchi disk of 20 cm diameter. In addition, turbidity was measured with a spectral fluorometer *AlgaeTorch 10* (bbe Moldaenke GmbH, Schwentinental, Germany) at the water surface and at a depth of 1 m during 1 min. This device measures chlorophyll-a [µg chl-a/l] from microalgae and cyanobacteria present in the water body, as well as turbidity in FTU (Formazine Turbidity Index) units. The measurements for secchi depth, turbidity, and chlorophyll-a content were taken twice at some sites, with 2 weeks apart. Here, the values were averaged and presented in Table 1.

The colored squares were lowered into the water with the help of a rope and a weight at 10–20-cm steps and photos were taken at each step with a GoPro Hero 5 or 7 Black action camera of 12 MP, with medium and linear field of view (FOV), respectively. Here, the camera was held directly underneath the water surface and 1 m above the water surface. The images were then analyzed by bare eye by 7 people to assess the maximum depth at which the color of the item, the shape of the item, and the item itself (irrespective of color or shape) of different sizes were visible (Fig. 2). The analysts received labeled images and instructions to carry out the evaluations. During analyses, the assessment was repeated for images with highest standard deviations to avoid methodological errors. Based on these results, the median of the maximum visibility depth for each item size was calculated for comparison between sites of different water transparency.

### Aerial drone experiments with floating items

Aerial experiments for floating items were carried out at three sites of different water transparency to assess how item detection accuracy varied water conditions (transparency, water color and bottom substrate), item characteristics (color and size), and flight height.

The experiments used a *DJI* Phantom 4 Pro V2 aerial drone coupled with a gimbal camera of 20 MP. The surveys were carried out in cloudy conditions (to avoid sun glint), no precipitation, and at wind speeds < 10 m/s. Sampling took place at three sites with *z*_SD_ = 1.2–5.8 m, namely Drewitzer See, Dorf Schmarl, and Gehlsdorf (Fig. 1, Table 1) to compare results under different water transparencies. PVC plastic squares of four sizes (2.5 cm, 5 cm, 10 cm, and 15 cm) and seven colors (white, black, grey, blue, green, yellow, and red) were used. These were attached to a net using metal wire or Velcro cable-ties, which allowed the plastic material and the net to float to simulate floating items (Fig. 2). The net was placed on the water at a site with ca. 1 m water depth. The seven colors were tested separately, including pieces of all sizes to determine a threshold for visibility. The number of plastic pieces varied per size, with higher number of smaller pieces. Single pictures of the floating net were captured with the drone camera at nadir position and automatic mode at 5 m, 10 m, and 20 m flight height from ground level, aspect ratio 4:3, resolution of 4864 × 3648, and focal length of 8.8 mm. The area covered by an image was 48 m^2^, 165 m^2^, and 690 m^2^ for each flight height respectively, with a maximum ground sampling distance of 0.62 cm/pixel. Coordinates of the site, bottom substrate, and water color are presented in Table 1. Water transparency was measured with the Secchi method.

### Aerial drone experiments with submerged items in shallow water

Aerial experiments for items under shallow water were carried out at two sites of different water transparency, namely Oldendorf and Drewitzer See with *z*_SD_ = 1.2 and 5.8 m (Fig. 1, Table 1) at shallow water depths of 0.5–1 m. The surveys were carried out using the same *DJI* Phantom 4 Pro V2 drone settings and similar weather conditions as for the floating experiment to assess how item detection accuracy varied with water conditions (transparency, water color, and bottom substrate), item characteristics (color and size), and flight height.

PVC plastic squares of 5 sizes (2.5 cm, 5 cm, 10 cm, 15 cm, and 20 cm) and 6 colors (black, white grey, blue, yellow, and green) were used which were fixed on a metal cross to lay firmly underwater on the ground (Fig. 2). A rope was attached to each cross to be able to regain them. The crosses were placed at three different water depths (35 cm, 65 cm, 100 cm). Colors were tested separately, including pieces of all sizes to determine a threshold for visibility. The number of plastic pieces varied per size, with higher number of smaller pieces. For each color and water depth, single images were captured at two flight heights: 10 m and 15 m from ground level, aspect ratio 4:3, resolution of 4864 × 3648, and focal length of 8.8 mm. At Drewitzer See, due to the high water transparency, surveys were carried out at 1 m and 1.7 m water depth and same flight heights. The area covered by an image was 165 m^2^ and 391 m^2^ for each flight height respectively, with a maximum ground sampling distance of 0.46 cm/pixel. Coordinates of the site, bottom substrate and water color are presented in Table 1. Water transparency was measured with the Secchi method.

### Underwater drone experiments with items on the seafloor

Underwater experiments for items on the seafloor were carried out at five sites of different water transparency and bottom substrate (sand, silt, algae, seagrass and mussel beds) with water depths between 3–11 m and *z*_SD_ = 1.8–5.8 m (Fig. 1, Table 1), to assess how item detection accuracy varied with water conditions (transparency, water color, and bottom substrate), item characteristics (color and size), and dive height.

The underwater drone BlueROV version 2 (*Blue Robotics, California, USA*) was used, which has a high definition camera (1080p, 30 fps) and four lights which serve to ease diving orientation and maneuver. The images were acquired by a GoPro Hero 5 or 7 action camera of 12 MP attached to the bottom of the ROV at nadir position. The experiments were conducted on sunny weather to allow for sufficient exposure to sunlight to ease visibility underwater.

Sampling sites were Groß Klein at the Warnow estuary and Stoltera, Hohe Düne, and Markgrafenheide at coastal waters, which were reached with the research boat *Klaashahn.* At the sites Dierkow and Schnatermann, no sampling was possible due to very low visibility and removal of silt during ROV operation which impeded taking pictures. In addition, experiments were also carried out at the lake Drewitzer See, with the help of a small pedal boat (Fig. 1). Sites were firstly inspected with the ROV to assess existing bottom substrate and underwater structures where litter could be trapped. Water depth was recorded from the sonar data on the boat or estimated with a weight attached to a rope. Water transparency was measured with the Secchi method.

PVC plastic squares of four sizes (2.5 cm, 5 cm, 10 cm, and 15 cm) and seven colors (white, black, grey, blue, green, yellow, and red) were attached to a net using metal wire or Velcro cable ties. This time the net was fixed to a metal frame of 150 × 60 cm, which was heavy enough to sink to the bottom to simulate items on the seafloor (Fig. 2). The seven colors were tested separately, including pieces of all sizes, to determine a threshold for visibility. The number of plastic pieces varied per size, with higher number of smaller pieces. Once the net was at the bottom, the ROV hovered above the frame and pictures were taken every 2 s on the time lapse mode with a linear or medium field of view (FOV), aspect ratio of 4:3, resolution of 4000 × 3000, and focal length of 3 mm, at a distance of 0.5 m up to 2 m. At Drewitzer See, a dive height up to 3 m was reached; however, for comparison only, dive heights of 0.5–2 m were considered for analysis. The area covered by an image was 1 m^2^, 4 m^2^, 6 m^2^, and 12 m^2^ for each dive height respectively at nadir, with a maximum ground sampling distance of 0.1 cm/pixel. Coordinates of the site and bottom substrate type are presented in Table 1.

### Item detection

Item detection analyses from the recovery experiments were carried out by manual image screening. The tool DotDotGoose (DDG) (Ersts, 2021) by Biodiversity informatics from the American Museum of Natural History is a free software used for tagging, counting, and categorizing items on images based on the choice of the evaluator and was used to facilitate the tagging and counting of items of different color and size. The analysts (8 for floating items, 5 for underwater items, and 6 for seafloor items) only received information on the color and sizes available but not on the number of squares per image and screened them without using the zoom function, starting with images from the lowest to the highest distance to the net/frame. Due to the irregular change of color tone in items underwater, based on water conditions (turbidity, color, transparency, depth) and bottom substrates, the DDG counting method was compared to item tagging on a regular pdf image, where analysts could label the color of underwater items themselves. Here, 13 analysts received a short training file but did not have any information on the number of items per image, neither whether more than one size or color were present per image. A data set of 27 random images (where the frame was fully visible) having different water transparencies and bottom substrates were analyzed, without using the zoom function.

### Estimation of accuracy and error percentage

Detection accuracy was defined as the mean number of squares detected versus the real number of squares per image. A threshold of high accuracy was set at 90%. Accuracy and error of detection were then determined as:

$$\begin{aligned} & \mathrm{Accuracy }\left[\mathrm{\%}\right]\hspace{0.17em} = \hspace{0.17em}100-[[\left|\left(\mathrm{mean\;items\;detected} \right. \right. \\ & \quad \left. \left. -\mathrm{Real\;number\;of\;items}\right)\right|] /\mathrm{ Real\;number\;of\;items}]\hspace{0.17em}\times \hspace{0.17em}100]\end{aligned}$$

$$\begin{aligned}& \mathrm{Error }[\mathrm{\%}]\hspace{0.17em}=\hspace{0.17em}[(\mathrm{mean\;items\;detected} \\ & \quad -\mathrm{real\;number\;of\;items}) /\mathrm{ real\;number\;of\;items}]\hspace{0.17em}\times \hspace{0.17em}100\end{aligned}$$

where a negative error value would be an error of omission (lower number of items detected than the real number) and a positive error value would be an error of commission (higher number of items detected than the real number). The reliability of both screening methods (pdf method and DDG) was estimated as the standard deviation from the total average detection accuracy per recovery experiment over all sites and conditions.

### Cost-efficiency

Official recommendations in the European Union (JRC, 2013) for the definition of a monitoring strategy require an estimation of effort and cost-efficiency. In our approach, we evaluate the cost-efficiency of two drone-based monitoring methods (one for floating litter and one for seafloor litter) in contrast to currently used methods, considering four coastal sites to be monitored four times a year, where litter items of all sizes shall be detected.

For floating litter, the UAV survey is compared to on-boat observation surveys. Here, we consider a line transect of 100 × 50 m in which images are taken at a flight height of 20 m (ca. 100 images with 80% image front and side overlap) and later analyzed by a researcher to estimate number and types of litter found. In the on-boat observation method, litter items are counted by two observers on a boat moving along the transect and annotations on litter type, size, and location are made in the moment. For seafloor litter, the ROV survey is compared to scuba divers. Here, images are taken in a transect of 100 × 4 m (area = 400 m^2^), calculated at camera field of view angle (45°) and dive height of 2 m. Images are later analyzed to estimate number and types of litter found along the transect. In the scuba diver method, the litter is collected along the transect and later analyzed to estimate item number, types and material.

The estimations of costs, time, and staff needed were based on own experiences in the Baltic Sea region and established protocols from JRC (2013) and UBA (in German Environmental Agency Report, in press), as well as current market values. Costs were divided into *Investment and Initial costs* for implementation, *Office costs* for organization and planning, and *Field and Lab costs* for the monitoring itself. *Investment costs* include the costs of devices and materials calculated according to the current market prices in Germany with a depreciation period of 3 years for the UAV and ROV drones and 10 years for diving material. *Annual replacement costs* for materials and equipment include the rental of a small research boat (6.5 m length, 3 m width; 200 € per day) for the on-boat observation, scuba diver survey, and ROV method as well as other materials that need annual replacement. *Initial costs* include the staff costs for the implementation period as well as a testing period for each method of 1–2 months. *Office costs* consider staff costs needed for organization and planning of monitoring, considering permissions, orders, reporting, etc. Likewise, *Field costs* consider staff costs needed for the monitoring itself, analysis, and documentation of results. Staff costs were estimated at 37.50 € per hour (salary for a state authority in Germany); however, hourly costs and staff numbers can be adapted to each local context. In this sense, the UAV field survey considered one person, the on-boat observation considered three persons (two observers and one boat driver), the ROV method considered two persons, and the scuba diver method considered five persons (four research divers and one boat driver). Analysis of data was estimated to require one researcher and additional help of volunteers for manual image screening. In contrast, the on-boat observation survey and scuba diver methods require only one researcher for post-processing and analysis of the data. Time estimated for each method considers travel time to and from sampling location, survey time and analysis time. The resulting total monitoring costs are scored as 1 (very high) > 100,000 €, 2 (high) 75,000–100,000 €, 3 (moderate) 50,000–75,000 €, 4 (low) 25,000–50,000 €, 5 (very low) 10,000–25,000 €, as suggested by the JRC (2013, modified).

The efficiency of the monitoring methods was evaluated based on four criteria: accuracy, reproducibility, flexibility, and quality. Accuracy refers to the amount of items found by the method in comparison to the real number of items. Reproducibility reflects the possibility of the same method being carried out by different people deriving the same result. Flexibility refers to the extent to which the method is dependent or not on external influences (weather, permissions, drone functioning). Finally, quality refers to the type and quality of data acquired by each method (i.e., type and number of items, type of material, and spatial distribution) and the conclusions that can be derived from it. Each of the criteria was assessed with the scores 1 (very low), 2 (low), 3 (moderate), 4 (high), and 5 (very high). The efficiency score was the average of all evaluations. Finally, cost-efficiency was obtained by the multiplication of the cost and efficiency scores, classified as < 5 (very low), < 10 (low), < 15 (moderate), < 20 (high), and > 25 (very high).

## Results

### Camera tests on visibility of items underwater

**Fig. 3:**
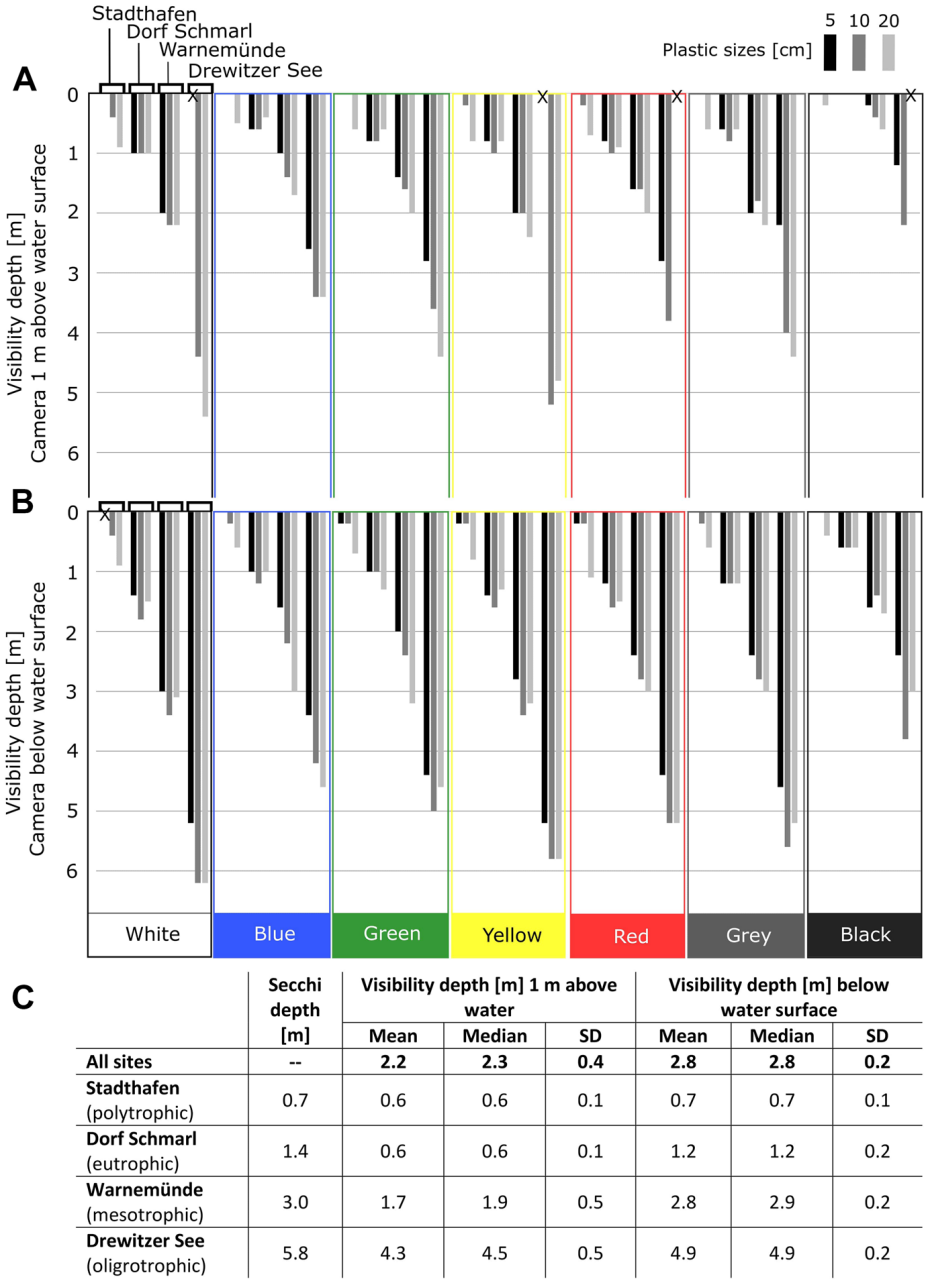
Median maximum visibility depth [m] of items of different sizes (5, 10 and 20 cm) and 7 colors. **A**. Visibility depth for images 1 m above water surface and **B**. right below water surface at sites of different water transparency: Stadthafen (polytrophic), Dorf Schmarl (eutrophic), Warnemünde (mesotrophic) and Drewitzer See (oligotrophic) (Fig. 1). **C**. Mean, median and standard deviation (SD) of maximum visibility depth [m] averaged for all colors. The assessment of visibility also considered the maximum depth of detection for each color and shape underwater. In general, visibility depth for color and shape were lower, for detailed results see Fig. S1 and S2

**Fig. 4:**
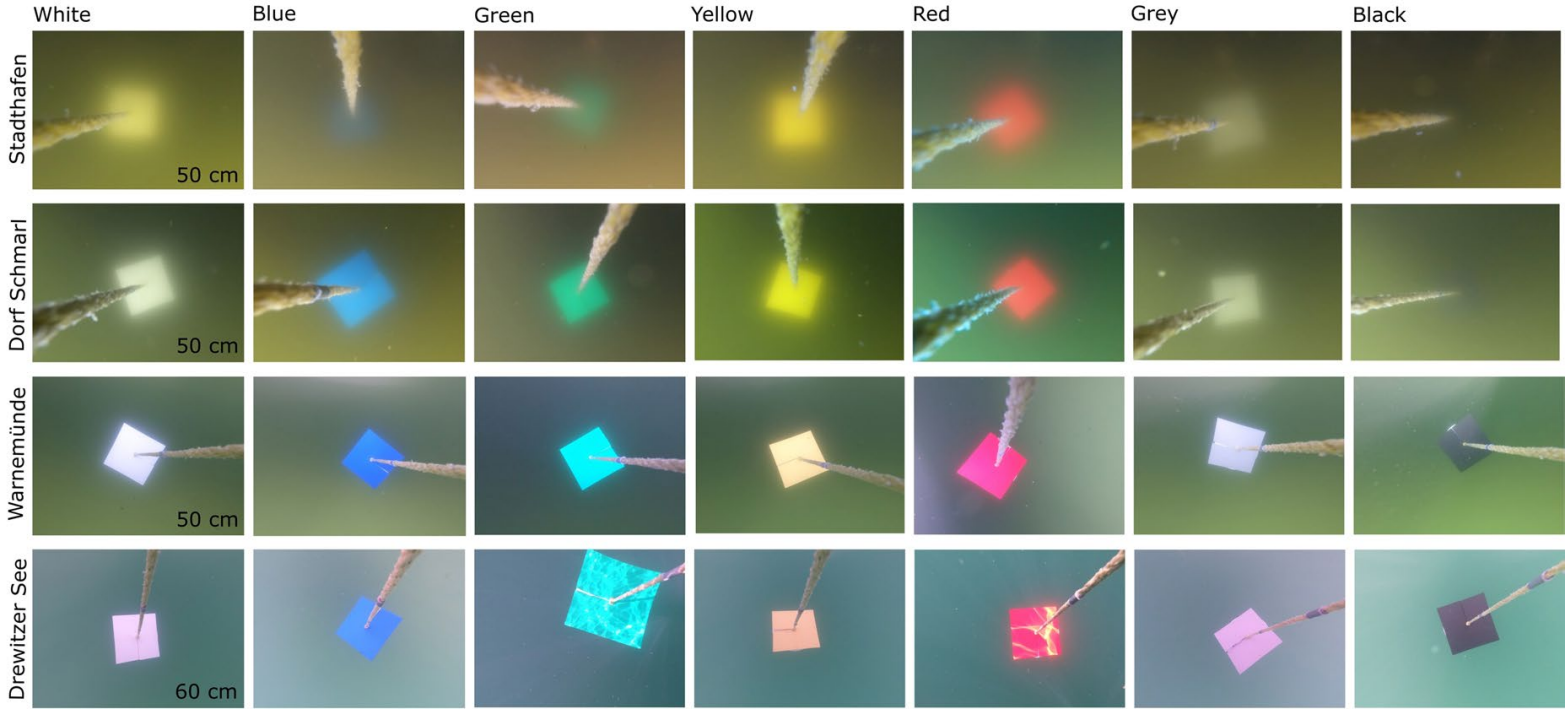
Examples of change in color tones and visibility at sites of different water transparency. Comparison of items at similar depth per site, during visibility tests

The images were evaluated by 7 people with standard deviations between 0.1 and 0.5 m for visibility depth averaged for all colors (Fig. 3C). The visibility depth of the items varied strongly with water transparency, with overall highest visibility depth in mesotrophic (Warnemünde) and oligotrophic (Drewitzer See) conditions (Fig. 3). A higher visibility depth of the items was achieved with the camera below water (B) than above water (A), by 0.1 to 2.4 m more depending on item size, color, and site conditions (Fig. 3). Waves and light reflections at the sea surface generally reduced the usability of photos taken above the surface, and thus reduced visibility of the items. In terms of color, the item colors most visible were white and yellow with visibility depths of up to 6.2 m and 5.8 m, respectively (Fig. 3B). In contrast, black was the color with least visibility with depths of 0–2.2 m for images taken above the water surface (A) and 0–3.8 m for images taken below the water surface (B) (Fig. 3). Colors were also subject to change of tone based on the water conditions, as it was the case with white, yellow, grey, and green (Fig. 4). Due to this, the visibility depth to recognize color (without changes in tone) and the item shape (square) was lower than the visibility depth to identify the item itself (Fig. S1 and S2). In terms of size, larger items were on average detected at a higher depth. Between items of 5 and 10 cm, a difference in visibility depth of 0–1.4 m (below water, B) and up to 1.8 m (above water, A) was seen. Between items of 10 and 20 cm, the difference in depth visibility was 0–0.8 m for images below water (B) and up to 1 m for images above water (A) (Fig. 3). Larger differences were seen with water transparency (e.g., Warnemünde, Drewitzer See); thus, this factor played the most important role in detection of submerged and underwater items, whereas item size and color played a minor role.

### Aerial drone experiments with floating items

The images were evaluated with the DDG counting tool by 8 people, reaching an overall item detection accuracy of 86 ± 13% averaged for all item sizes, flight heights, and sites.

#### Detection of item size

**Fig. 5:**
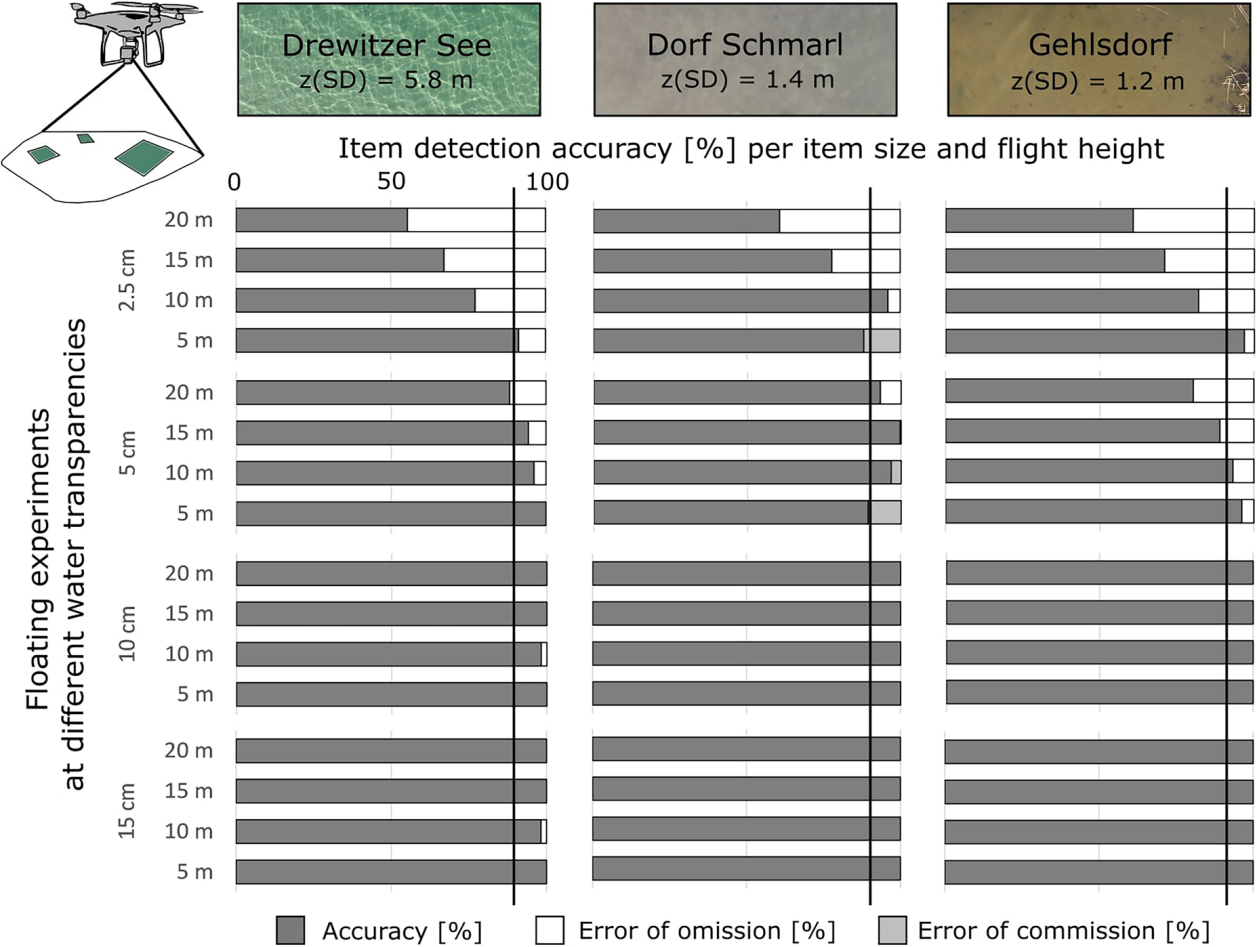
Accuracy and error of detection [%] for item sizes 2.5 cm, 5 cm, 10 cm and 15 cm, per flight height for the floating recovery experiment at sites of different water transparency

Detection accuracy for floating items of different size was in most cases > 90% and did not differ largely between sites (Fig. 5). Main differences were seen for item sizes and flight heights. When aggregated for flight heights, items of 2.5 cm had lowest detection accuracy (73–81%), in contrast to items of 5 cm, 10 cm, and 15 cm items (> 90%) (Fig. 5). Increasing flight heights led to a decrease in detection accuracy by ca. 40% between images taken at 5 m versus 20 m. This was observed only for items of 2.5 cm and 5 cm, whereas for items > 10 cm flight heights did not play a relevant role (Fig. 5). Error of omission (missing items) occurred at all sites mainly for items of 2.5 cm and 5 cm, with 3–45% error. Commission errors (adding items) occurred at lowest flight height (5 m) at the site Dorf Schmarl for items of 2.5 cm and 5 cm (Fig. 5). This may have occurred due to presence of leaves and foam on the water surface that may have been confused and counted as the plastic item.

Factors influencing the detection of floating items were mainly item size and flight height, whereas water transparency did not play a relevant role. In this sense, items > 5 cm can be detected with > 90% accuracy at flight heights up to 20 m.

#### Detection of item color

**Fig. 6:**
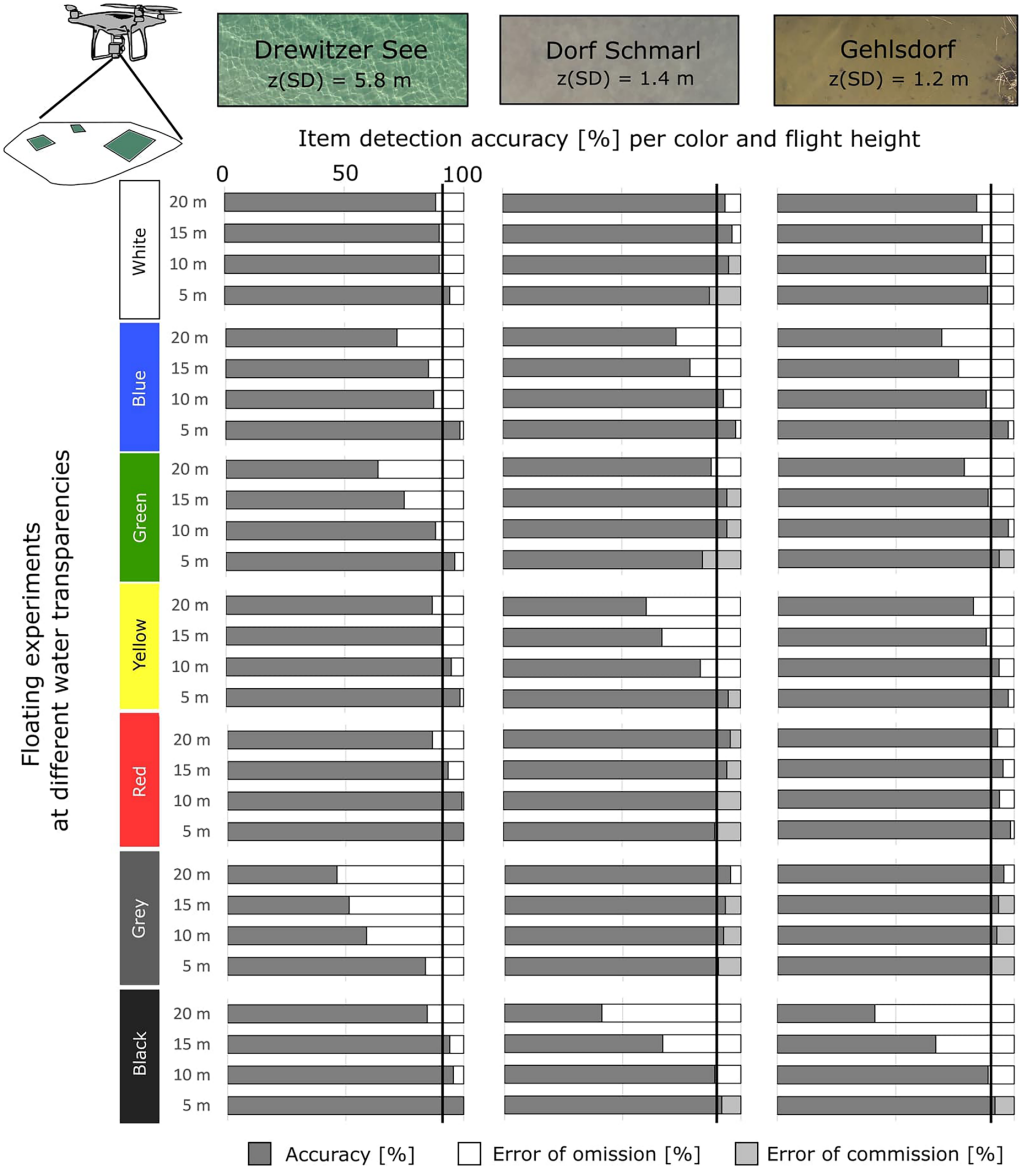
Accuracy and error of detection [%] for each color per flight height for the floating recovery experiments at sites of different water transparency

Detection accuracy for floating items based on colors was in general > 90%. Red was on average the color best detected (> 90%) in all sites, followed by white, green, and yellow which presented accuracies of 76–93% (aggregated for flight heights). At the oligotrophic site (Drewitzer See), white, yellow, red, and black were detected with highest accuracy (85–100%). Grey, blue, and green showed lower detection accuracy than other colors (46–99%) (Fig. 6). At eutrophic sites (Dorf Schmarl and Gehlsdorf), red, grey, green, white, and yellow were detected with > 83% accuracy (Fig. 6).

The detection of item color decreased with flight height at all sites, but this pattern was more clearly visible for blue, green, yellow, grey, and black, whereas white and red seemed to be detected at similar high accuracies at all flight heights and sites (Fig. 6). Error of omission occurred for all item colors, however varied with water transparency and water color of each site. Colors with highest errors of omission were grey (at Drewitzer See), yellow (at Dorf Schmarl), and black (at Dorf Schmarl and Gehlsdorf), with values between 27 and 40%. Errors of commission were mostly present at Dorf Schmarl for white and green (ca. 9% each) (Fig. 6).

In a set up where only items of 2.5 cm of all colors were presented at once, detection accuracies were similar as for the first set up where items of different sizes and one color at a time was shown. However, it is clearly visible that white and red were better detected in comparison to other colors (Fig. S3).

In this sense, item color and its contrast to the water color seemed to be a more important factor in the detection of items than water transparency.

### Aerial drone experiments with submerged items in shallow water

The images were evaluated with the DDG counting tool by 5 people, reaching an overall item detection accuracy of 64 ± 17% averaged for all item sizes, flight heights and sites.

#### Detection of item size

**Fig. 7:**
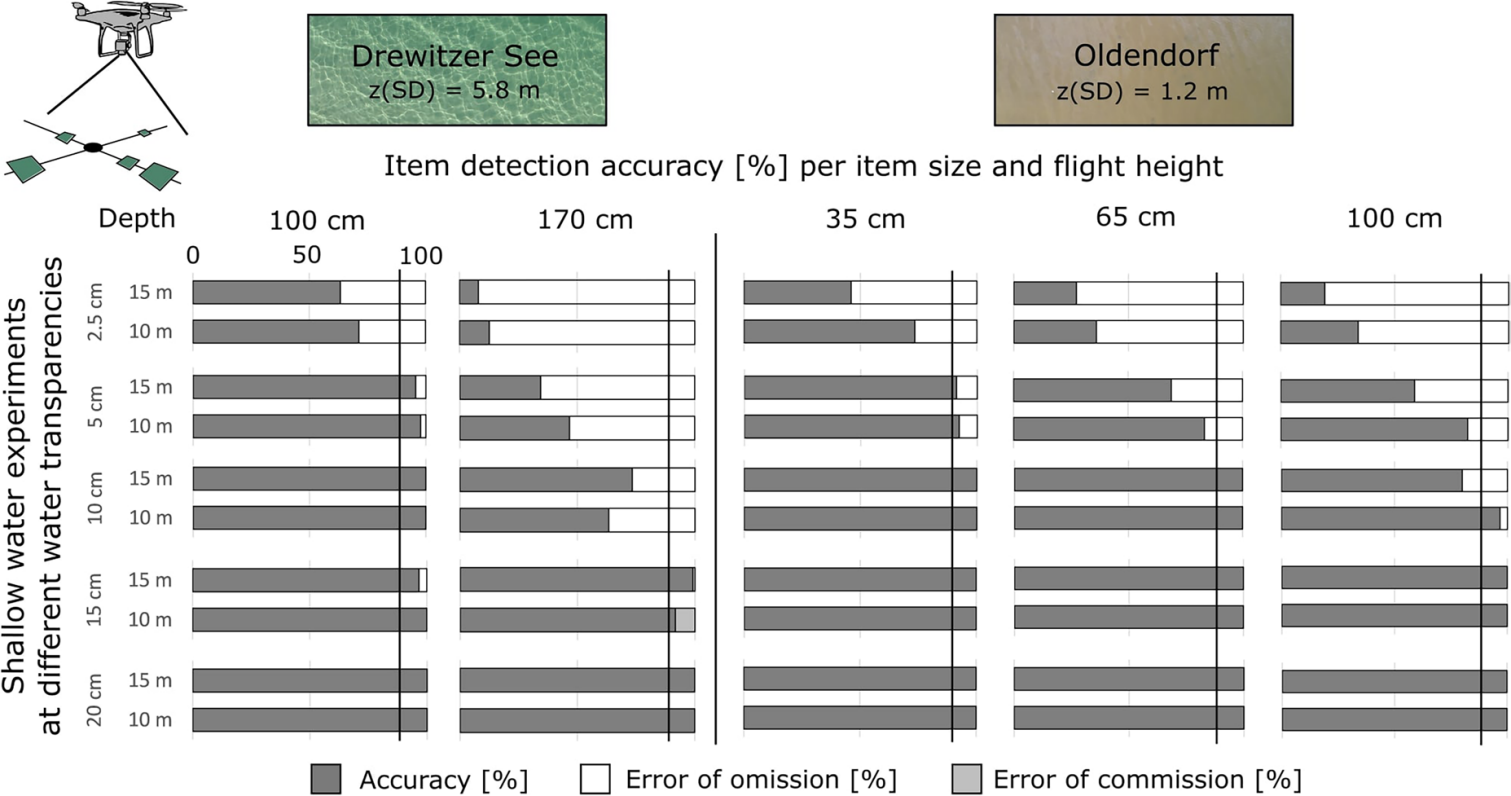
Accuracy and error of detection [%] for item sizes 2.5 cm, 5 cm, 10 cm, 15 cm and 20 cm, per flight height for the recovery experiments of submerged items at sites of different water transparency

Due to very different water transparencies at the sites, water depths for recovery experiments were adapted. Here, we focus on the comparison of results at 1 m water depth. Water transparency was a factor that influenced the detection of submerged items, with higher detection accuracy at the oligotrophic site (Drewitzer See) for items of 2.5 cm with 63–71% versus 19–34% at the eutrophic site (Oldendorf) and items of 5 cm with 96–98% versus 59–82%, respectively (Fig. 7). Increasing flight heights led to a decrease in detection accuracy of submerged items by 1–27% for images taken at 10 m versus 15 m. Similarly, increasing water depths by 30 cm led to a decrease in detection accuracy by 10–30% at the eutrophic site (Oldendorf), while at the oligotrophic site (Drewitzer See) a 70-cm water depth difference led to 0–61% lower detection accuracy (Fig. 7). These differences were mainly seen for items < 10 cm. Error of omission occurred mainly for item sizes < 5 cm, in both sites. Error of commission occurred in only one case for items of 15 cm at lowest flight height at Drewitzer See (Fig. 7). Threshold for high accuracy (> 90%) was thus reached for items of 10 cm, 15 cm, and 20 cm at both sites and items of 5 cm could be well detected at sites of high water transparency. In this sense, submerged items > 10 cm can be accurately detected from the air with minimum influence from water transparency, at flight heights of up to 20 m and water depths of 1–1.7 m (< 10 cm).

#### Detection of item color

**Fig. 8:**
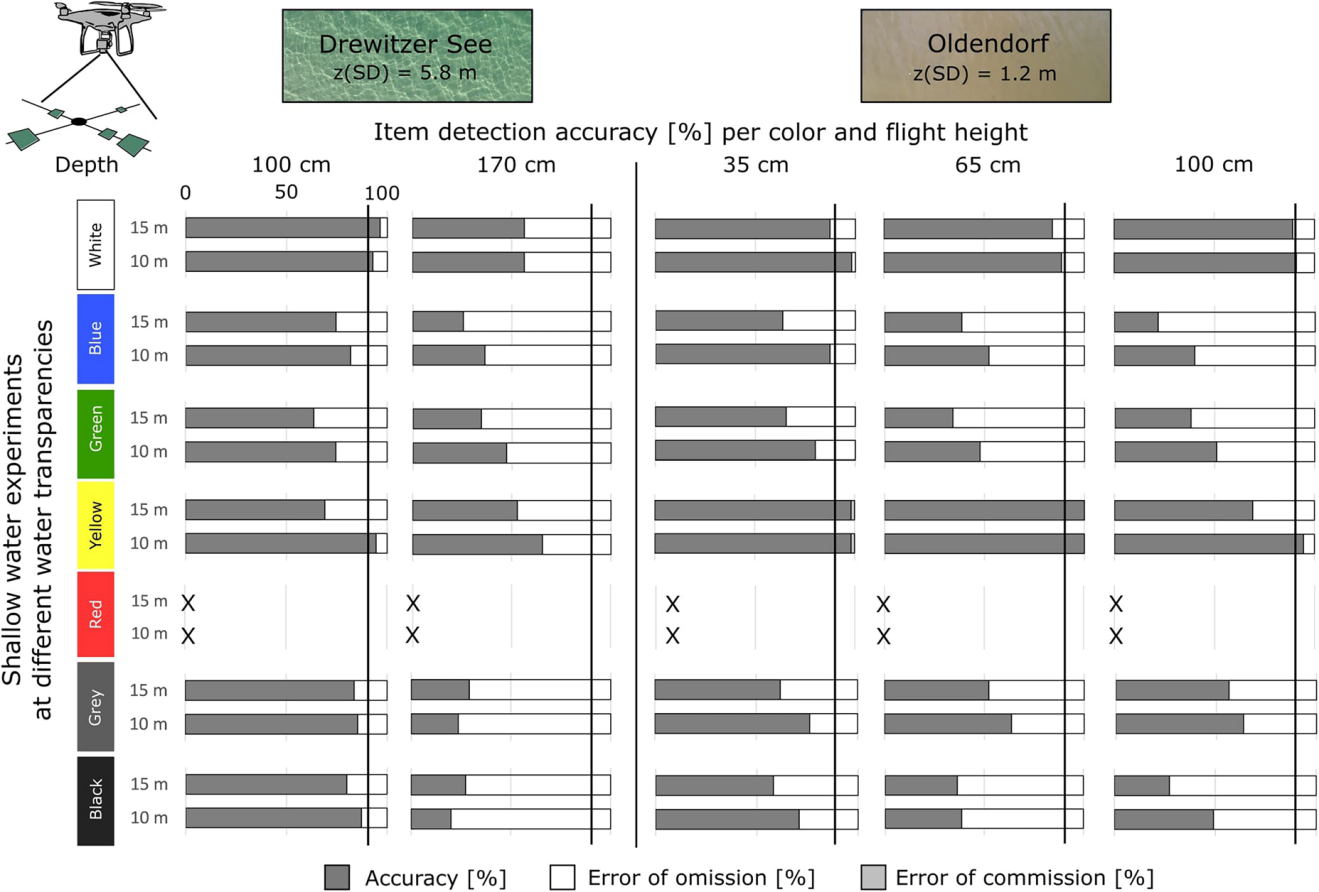
Accuracy and error of detection [%] for each color per flight height for the recovery experiments of submerged items at sites of different water transparency

Due to differences in water depth, we mainly compare the results from 1 m water depth for both low and high water transparency sites (Fig. 8). Red was not tested in this experiment.

Only white and yellow reached the threshold of high accuracy (> 90%). Other colors like blue, green, grey, and black presented detection accuracies > 75% at the oligotrophic site (Drewitzer See) (Fig. 8). When comparing same water depth and item colors, it becomes evident that water conditions (transparency and color) of each site influenced detection accuracy of submerged items. Increasing water depth decreased detection accuracy by 10–50% for ca. 70 cm difference at both sites, except for white and grey at the eutrophic site (Oldendorf) which maintained similar accuracy (Fig. 8). The detection accuracy of item color also decreased with higher flight heights, 10–30% less at 10 m versus 15 m flight height (Fig. 8). In this sense, both water depth and flight height influenced detection accuracy in a similar way; however, water transparency and water color played a more relevant role.

### Underwater drone experiments with items on the seafloor

The images were evaluated with the DDG counting tool by 6 people, reaching an overall item detection accuracy of 78 ± 21%. A separate pdf method was used for comparison, reaching an overall accuracy of 57 ± 34%, averaged for all item sizes, dive heights, and sites.

#### Detection of item size

**Fig. 9:**
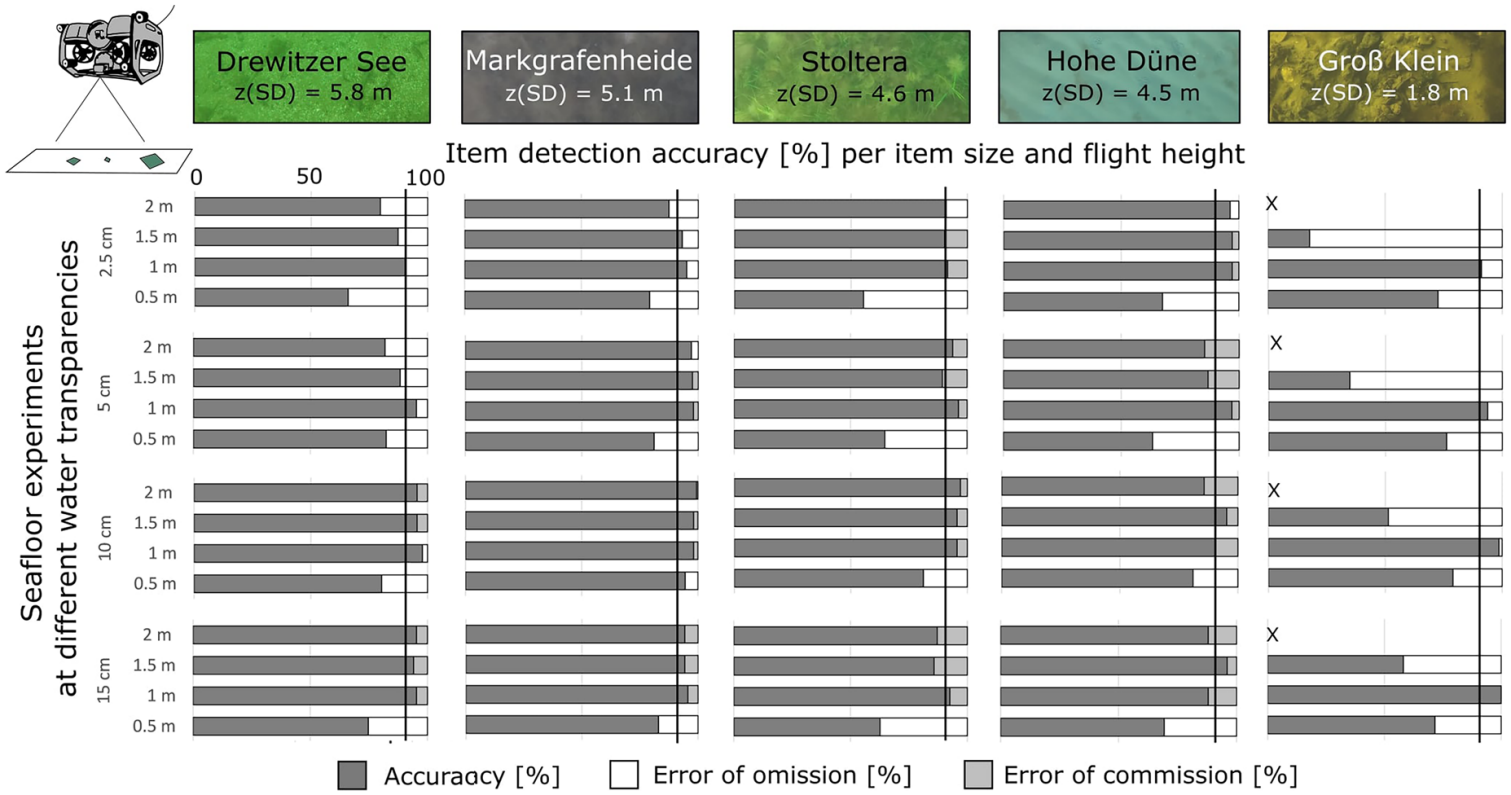
Accuracy and error of detection [%] for item sizes 2.5 cm, 5 cm, 10 cm and 15 cm, per dive height for the recovery experiments of underwater items at sites of different water transparency and bottom substrate

Detection accuracy for items on the seafloor was very similar regardless of item size. Slightly higher detection accuracy was observed for items of 10 cm and 15 cm; however, main differences were observed between sites of highest and lowest water transparency and different bottom substrate (Fig. 9). Mesotrophic and oligotrophic sites (*z*_SD_ = 4.5 m–5.8 m), namely Hohe Düne, Stoltera, Markgrafenheide, and Drewitzer See (Table 1), reached > 90% for all item sizes (Fig. 9). In contrast, the eutrophic site (Groß Klein, *z*_SD_ = 1.8 m) showed accuracies 10–60% lower (Fig. 9). Error of omission occurred at all item sizes and sites. Error of commission occurred mainly at sites Stoltera, Hohe Düne, and Markgrafenheide with values of 10–15% (Fig. 9) with seagrass, sand, and mussels as bottom substrates, however having similar water transparency (Table 1).

In terms of ROV settings, lower accuracy was observed at 0.5 m dive height (also having higher omission error) and was higher at 1–2 m (Fig. 9). This may be due to lack of visibility of the entire the frame and experimental errors in the counting of items. At Groß Klein, lower accuracy was seen at 1.5 m dive height (Fig. 9), likely due to low water transparency. In this sense, item size played a minor role in underwater item detection whereas water transparency and bottom substrate played a more relevant role due to the contrast of items with the background.

#### Detection of item color

**Fig. 10:**
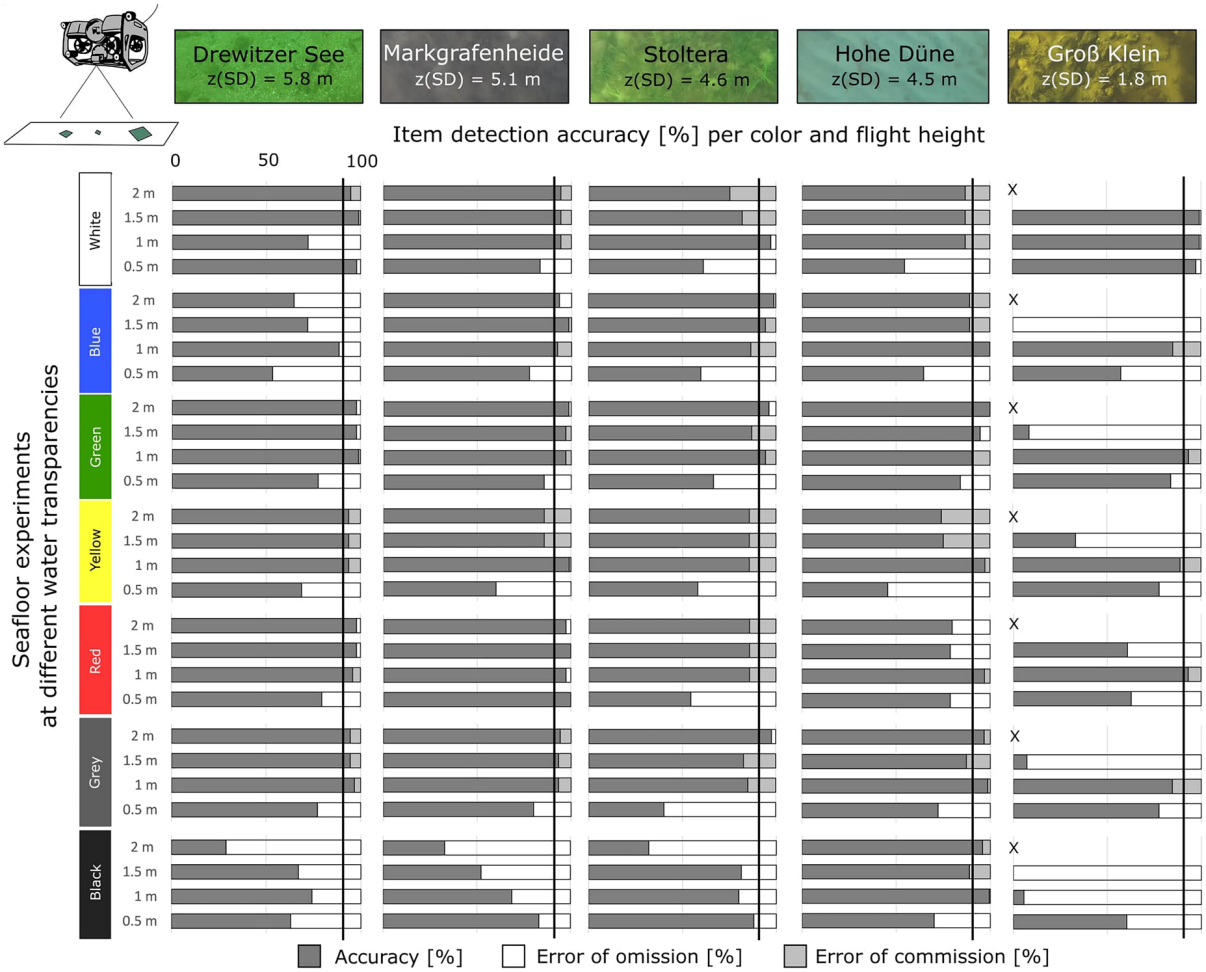
Accuracy and error of detection [%] for each color per dive height for the recovery experiments of underwater items at sites of different water transparency and bottom substrate

Using the DDG counting tool, > 90% detection accuracy was reached at all sites (Fig. 10). The colors with overall highest accuracy were white, green, and grey, with slight differences between levels of water transparency and different bottom substrate. At the oligotrophic site (Drewitzer See), white, green, yellow, red, and grey had accuracies > 90%, while at Markgrafenheide (mesotrophic), blue also had > 90% accuracy. Mesotrophic sites, Stoltera and Hohe Düne, presented similar detection accuracies for most colors. At the eutrophic site Groß Klein (site of lowest water transparency), white, green, and red reached > 90% detection accuracy. Black was in general the color least detected, except at Hohe Düne (Fig. 10). Errors of omission and commission occurred for all item colors but depended on site conditions. At Groß Klein, all colors except for white presented high omission error (3–100%), whereas at Stoltera, yellow and red; at Hohe Düne, white and yellow and at Markgrafenheide, yellow and black presented high omission errors (> 40%). In contrast, at Drewitzer See, black presented highest omission error (25–71%). Commission errors were highest (> 15%) at Stoltera and Groß Klein, especially for white, blue, and grey (Fig. 10). This means that not only water transparency was a factor that influenced the detection accuracy of item colors, but also the contrast to water color and bottom substrate. In terms of ROV settings, lowest detection accuracy was observed at the lowest dive height (0.5 m) and in most cases similar accuracy was reached for dive heights between 1 and 2 m.

The pdf counting method showed that the perception of colors varied between the evaluators (Table 2A). Blue, yellow, and white were the colors best identified (in > 70% of evaluations), whereas grey and black were the least correctly identified in only 15% and 13% of the evaluations, respectively. Black could “not be clearly identified” or was “not seen” in 49% and 23% of the evaluations, respectively. Green was often confused with blue in 41% of the evaluations. Similarly, grey was confused with white in 46% of the evaluations and yellow with white in 15%. Red was correctly detected in 62% of the evaluations, however also not clearly identified in 15%. Other colors that were mentioned were turquoise which was confused with green and pink. Similarly, evaluators saw more than one color in images where only white and grey items were present (Table 2A).

**Table 2:**
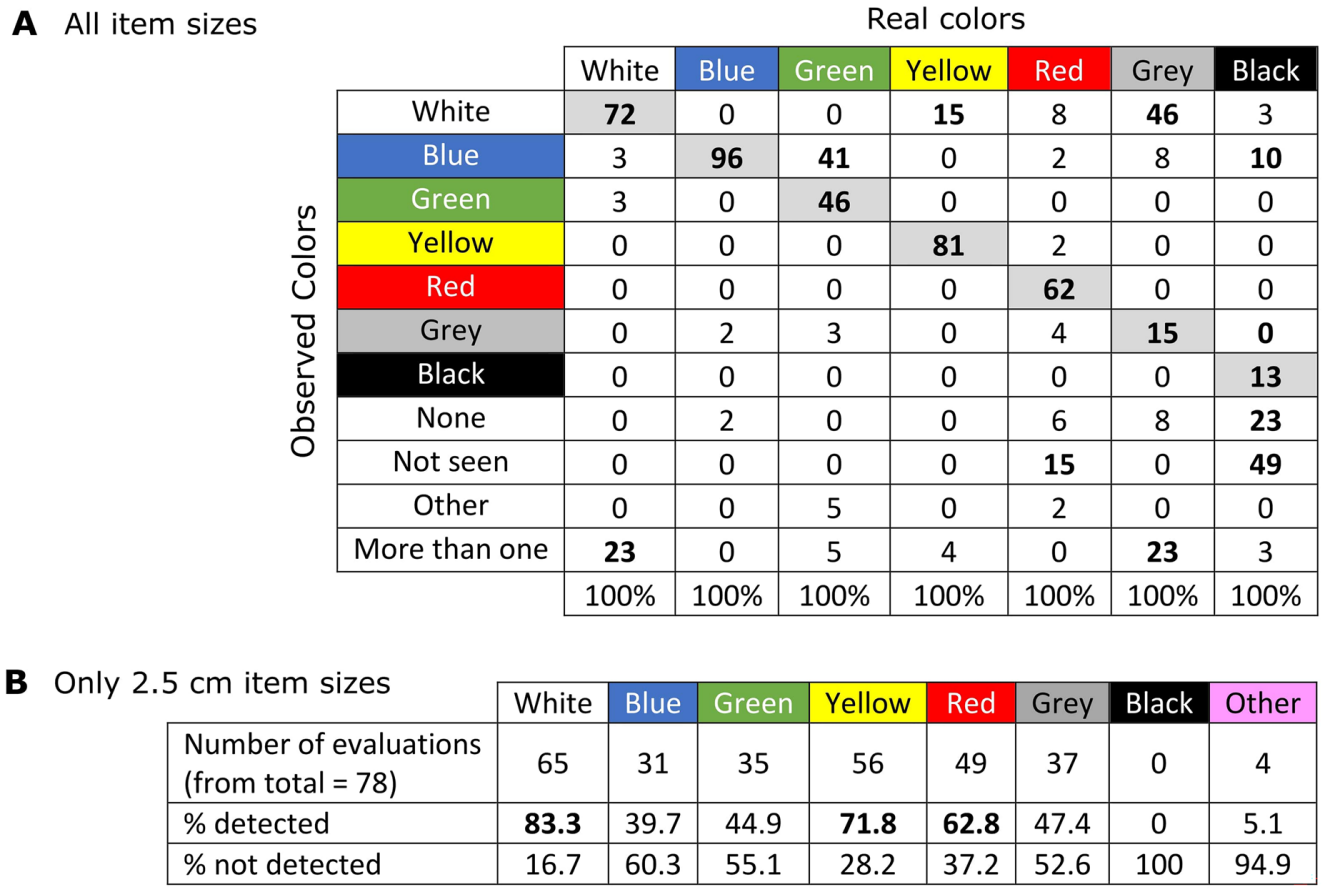
A. Detection of item color underwater with all item sizes using the pdf method. Per image, only one color was shown. Numbers are the percentage of evaluations that wereassigned per color. In bold, the evaluations with highest percentage per color. B. Detection of item color underwater only with items of 2.5 cm using the pdf method. Here, all colors were presented at once in one image. In bold, the colors with highest percentage of detection

In a set up where only items of 2.5 cm of all colors were presented at once, detection accuracies with the DDG method were similar as for the first set up where items of different sizes and one color at a time was shown, except for black where accuracies were ca. 50% lower at all sites (except for Stoltera) (Fig. S4). Whereas in the pdf method, white, yellow, and red were the colors best identified (> 70%) for items of 2.5 cm (Table 2B). In contrast to images where only one color was shown blue was only identified in 40% of the evaluations (in contrast to 96%), similar to green and grey. The latter was identified in 47% of evaluations in contrast to 15% as in the previous assessment. Finally, black was not identified in any of the images, and other colors such as “multicolor” or turquoise were also mentioned (Table 2B).

### Cost-efficiency

The average knowledge of the evaluators based on a self-assessment was 2.2 ± 0.8 in the ML monitoring aspect and 2.4 ± 0.5 in the drone implementation aspect (with 1: low, 2: medium, and 3: high).

Comparing monitoring methods for floating ML, overall costs and staff effort were very similar between the on-boat observations and the UAV drone method (Table 3). Equipment and material costs, including boat rental and drone purchase, were very similar between methods. Implementation time for the UAV drone method was estimated to be higher due to the need of testing and calibration of both sampling and analysis methods (e.g., algorithms for object detection methods, libraries for deep learning, or training of reviewers for image screening). Likewise, office costs were estimated to be higher for the UAV drone method due to the need of flight permissions which require more administrative work. In terms of field work and analysis effort, the on-boat observation was estimated to require more time in the field, whereas the UAV method was estimated to require less time in the field but more time for analysis. The total annual costs for monitoring of floating litter with the UAV method were 26,289 € for a monitoring of four sites, four times a year (1,643 € for single survey) versus 22,250 € (1,391 € for single survey) for on-boat monitoring. Costs between both methods were very similar, with on-boat survey falling into the very low (1000–25,000 €) and UAV method into the low (25,000–50,000 €) cost category. In the evaluation of efficiency, both on-boat observations and UAV share similar scores, with slightly higher scores for accuracy and quality and slightly lower in reproducibility and flexibility in the UAV method. In the final cost-efficiency score, both methods fall into the moderate cost-efficiency category: with 14.5 versus 12.1 score, respectively (Table 3).

**Table 3:** Cost-efficiency assessment for monitoring for floating and benthic marine litter, comparing drone vs. current methods. The values are based on own experience in the Baltic Sea region and established protocols from JRC (2013) and UBA (in German Environmental Agency Report, in press), taking into account the MSFD guidelines (JRC, 2013) and federal state authority staff salaries (37.50 € per hour) for a monitoring at four coastal water sites, four times a year. In bold are shown the scores for cost and efficiency, giving the cost-efficiency score

		**Floating marine litter**		**Shallow seafloor litter**	
		**On-boat**	**UAV**	**Scuba divers**	**ROV**
Investment and Initial costs	Equipment, tools, and their annual replacement costs	3,300.00 €	2,038.63 €	7,234.23 €	5,919.40 €
	Implementation time (hours)	200	266.67	200	266.67
	Total initial costs	7,500.00 €	10,000.00 €	7,500.00 €	10,000.00 €
Office costs	Staff effort (hours)	133.33	200	266.67	200
	Total office costs	5,000.00 €	7,500.00 €	10,000.00 €	7,500.00 €
Field and lab costs	Staff effort field (hours)	156	36	560	104
	Staff effort analysis (hours)	16	144	64	144
	Total field and lab costs	6,450.00 €	6,750.00 €	23,400.00 €	9,300.00 €
**Final costs**	**Total annual running costs**	14,750.00 €	16,288.63 €	40,634.23 €	22,719.40 €
	**Total annual costs**	22,250.00 €	26,288.63 €	48,134.23 €	32,719.40 €
	**Total hours**	505	647	1091	715
	**Score costs**^a^	**5**	**4**	**4**	**4**
Efficiency	Accuracy	3.0	3.5	4.2	2.9
	Reproducibility	3.2	3.0	3.4	3.0
	Flexibility	2.8	2.4	2.4	3.0
	Quality	2.6	3.2	4.6	3.2
	**Score efficiency**^b^	**2.9**	**3.0**	**3.7**	**3.0**
**Cost-efficiency score**^c^		**14.5**	**12.1**	**14.6**	**12.1**

^a^Costs score: 1 (very high) > 100,000 €, 2 (high) 75,000–100,000 €, 3 (moderate) 50,000–75,000 €, 4 (low) 25,000–50,000 €, 5 (very low) 10,000–25,000 €, as suggested by the JRC (2013, modified)

^b^Efficiency score: 1 (very low), 2 (low), 3 (moderate), 4 (high), and 5 (very high)

^c^Cost-efficiency score: < 5 (very low), < 10 (low), < 15 (moderate), < 20 (high), and > 25 (very high)

Comparing monitoring methods for benthic litter, overall costs and staff effort were higher for the scuba diver method than for the ROV drone method (Table 3). Equipment and material costs were slightly higher for the scuba diver method; however, implementation time was estimated to be higher for the ROV method to consider testing and calibration of sampling and analysis methods (e.g., algorithms for object detection methods or training of reviewers for image screening). Office costs were estimated to be higher for scuba divers, which require organizational time as well as potential training when involving volunteers. In terms of field work and analysis effort, scuba divers require more time in the field than a ROV method, whereas the ROV method requires more time for analysis. The total annual costs for monitoring of benthic litter with the ROV method were 32,719 € for a monitoring of four sites, four times a year (2,045 € for single survey) versus 48,134 € (3,008 € for single survey) for scuba diver method, ca. 15,000 € higher, with also ca. 380 h extra required in contrast to the ROV method. Still, both methods fall into the low (25,000–50,000 €) cost categories. In the efficiency assessment, divers were rated with a higher accuracy, reproducibility and quality than the ROV method. Contrary to this, the ROV methods appears to be slightly more flexible than the diver method. Considering cost and efficiency scores, both methods fall under the moderate cost-efficiency score, with 14.6 versus a 12.1 score, respectively (Table 3).

## Discussion

### Influence of water conditions, item characteristics, and method settings in the detection of floating, submerged, and seafloor litter

Water transparency was the main factor deciding whether monitoring can take place on a site. This was important for both the operation of the equipment and visibility of items. Results showed that low water transparency and dark water color such as in eutrophic sites (Dorf Schmarl and Gehsldorf) facilitated the detection of floating items due to high contrast, whereas high water transparency and clear water color such as in oligotrophic sites (Drewitzer See) would lead to more errors in detection of floating items, but higher detection accuracy for submerged and underwater items (Fig. 3). In this sense, the application conditions for both methods do not overlap 100% and should be considered according to the aim of study.

For floating items, the threshold for high accuracy (> 90%) was reached for items > 5 cm. However, items > 10 cm were detected with high accuracies at 20 m height (Fig. 5). Red was on average the color best detected at all sites, followed by white, green, and yellow (Fig. 6). Items of 2.5 cm showed best detection in colors red and white (Fig. S3). Here, item color and its contrast to the water color, as well as item size were more important for detection than water transparency. Thus, accumulation hotspots with litter items of larger size may be easily detected and differentiated from, e.g., birds or organic material based on size, shape, and color.

In contrast to detection of ML items on beaches, the detection of items floating in seawater has proved more accurate, likely due to the more homogenous background (Andriolo et al., 2022). Common limitations in remote sensing images of floating items are sun glint, cloud cover, bottom reflection, and other floating objects that may be confused with litter (Topouzelis et al., 2021). This was also observed by Atwood et al. (2018) who found best classification accuracy at sites with medium and high debris density and lower at sites with higher vegetation which disturbed classification. To cope with these disturbance factors, near infrared (NIR) and thermal infrared (TIR) sensors coupled to a UAV have proved successful at detecting floating plastics in these conditions (Goddijn-Murphy et al., 2022). Nevertheless, various automatic object recognition techniques have been suggested for RGB imagery, such as color difference image processing (Kataoka & Nihei, 2020) and RGB data is still suggested as more convenient in terms of data quality and budget (Pichel et al., 2012).

As soon as items start sinking, as for submerged items, water transparency and water depth were two influencing factors for detection with aerial drones. Here, items > 10 cm were best detected (> 90%) at sites of low and high water transparency and items of 5 cm could be well detected at the oligotrophic site (Fig. 7). The colors best detected were white and yellow at both sites in similar proportion; however, other colors were better detected at the site of highest water transparency. Here, red was not tested (Fig. 8). The experiment on item visibility underwater revealed that visibility was less with the camera above the water surface due to disturbance of waves and light reflections above water (Fig. 3); thus, the application of aerial drones for submerged items should consider this optical effect and carry out sampling on cloudy and non-windy weather conditions.

For items on the seafloor, water transparency and bottom substrate were the main determinants for item detection and item size played a minor role. The threshold for high accuracy (> 90%) was reached at oligotrophic and mesotrophic sites (Fig. 9). The colors best detected were white, green, and grey; however, it was highly dependent on the water transparency and bottom substrate at each site. Black was in all cases the one with lowest detection accuracy (Fig. 10), which was also the case when comparing color detection in items of 2.5 cm (Fig. S4). When comparing sites with similar water transparency but different bottom substrate (e.g., Stoltera, Hohe Düne, and Markgrafenheide), the latter seemed to be a more important factor in detection accuracy (Figs. 9 and 10). However, it remains unclear whether a comparison of sites with the same bottom substrate but different water transparency would lead to the same conclusion. Since the operation of the equipment requires high water transparency, it is necessary to consider this during the selection of monitoring sites. Underwater items may be easily confused with underwater structures (plants, rocks, mussels) when the color and shape is similar; thus, the identification of specific colors and shapes underwater can be an important factor to consider.

Items on the seafloor can be present in a variety of colors; however, colors are subject to change with light attenuation (i.e., loss of intensity) and distance underwater (Bazeille et al., 2012; Bianco et al., 2015), and as seen during the evaluation, color perception also varied between evaluators (Table 2). The changes in colors will also vary in more turbid water environments, since light absorption and scattering increase in the presence of algae, suspended organic and inorganic matter, and dissolved organic compounds (Bazeille et al., 2012). The visibility of both color and shape in our study decreased with depth faster than for items itself (Fig. 3 versus Fig. S1 and S2). Taking into consideration the colors that are most predominant in underwater habitats, i.e., green (vegetation), blue/green (water color shades), grey (stones), black, white (mussels), and yellowish/grey (sand), the most contrasting color for detection is red, which could serve as indicator for the presence of litter underwater and which had a visibility depth of up to 5 m (Fig. 3). Automatic detection methods underwater are rather based on object shape recognition; however, it is recognized that the use of both shape and color could derive more robust results (Bazeille et al., 2012). Our study considered only one shape (square), and thus conclusions cannot be given in comparison to other shapes. Nevertheless, detection of items only by color or shape result difficult and should be complemented with item size.

We observed a clear trade-off between flight/dive height and item visibility that was also observed in previous studies (Garcia-Garin et al., 2020a; Geraeds et al., 2019; Lo et al., 2020). We did not test item visibility and detection accuracy of larger items (> 20 cm); thus, the threshold for flight/dive height for visibility was very low (20 m/ 2 m) and focused on the item sizes majorly found in the shallow coastal environment (JRC, 2013). The optimal flight height for detecting floating items with an UAV in our study was at 15 m for items > 5 cm and 20 m for items > 10 cm. For submerged items, items > 10 cm could be detected with an UAV at 15 m with minimum influence from water transparency and water depth, in contrast to smaller items. Geraeds et al. (2019) determined that a flight height of 8–18 m above water level was sufficient to detect litter; however, a flight height of 4–6 m was needed to classify items. Similar constraints were observed in the study of Garcia-Garin et al. (2020a), who compared UAV surveys to vessel observation and concluded that although litter count is comparable between both methods, classification of the litter is limited with the UAV due to higher flight height of observation (65 m). Another study carried out at the beach investigating the effect of drone settings (flight height) and environmental factors (weather, time of day, and beach substrate) for item detection found that flight height was the only factor that influenced detection accuracy, with the lowest flight height (5 m) reaching highest accuracy, in contrast to the highest (15 m). Similarly, larger item sizes (30–50 cm) were better detected than smaller items (2.5–30 cm), detection of colors was lower with increasing flight height, and was influenced by beach substrate (Lo et al., 2020). We observed a high level of commission errors at lowest flight height (5 m) at the site Dorf Schmarl for items of 2.5 cm and 5 cm (Fig. 5) likely due to confusion with non-plastic items. In our study, the optimal dive height for items on the seafloor was between 1 and 2 m from the bottom. To our knowledge, previous studies have not discussed the requirements for item detection underwater nor the optimal distance to the target for detection. However, we suggest that a similar trade-off occurs, however also influenced by water turbidity.

### Manual image screening and deep learning approaches

Manual screening of images is a common and crucial approach to analyze RGB images obtained by aerial drones, since this provides not only a simple technique to assess ML abundance, distribution and hotspots in an environment, but it also provides the ground truth data for machine learning algorithms (Andriolo et al., 2021). It is recommended that at least 2–3 persons inspect images to reduce observer bias, however, considering more than one analysts is often restricted by the large amount of images to analyze, budget, or experience (Garcia-Garin et al., 2020a, b).

Our study included 5–8 trained volunteers for manual image screening, using a software for item tagging and counting (DDG) as well as a pdf method. For the analysis of items on the seafloor, mean detection accuracy and standard deviation was 78 ± 21% and 57 ± 34%, for each method respectively. Underwater images were particularly difficult to evaluate due to low water transparency, high suspended sediments, or complex bottom substrate, which may have led to stronger differences between the evaluators and thus higher standard deviations from the mean (in contrast to the other experiments), which may also depend on observation time, eye sight, patience, fatigue, and color perception from each person.

A similar study investigated the level of agreement between analysts in the identification of ML on beached items through manual image screening, considering different experiences, background and expertise. They found highest level of agreement between analysts with high expertise in UAV and litter monitoring (0.77–0.91 from a scale of 0 to 1), but observer bias was also influenced by the common type of items seen in their territories, with large differences in classification of items (Andriolo et al., 2021). Trained non-expert volunteers can also be considered through, for example, citizen science approaches, however reaching limited quality of results. Merlino et al. (2021) found that trained students were able to detect only 50% of the items observed in field studies, mainly due to an underestimation of small litter sizes (5–15 cm) and overestimation of fragments; however, the classification of litter followed similar proportions to the ground truth data. Red was the color that was best detected, and lower accuracy was found in colors that had lower contrast to the background (Merlino et al., 2021). In our study, red was the color best detected in floating items and white and yellow in submerged items, whereas the detection of colors for items underwater varied largely upon water conditions (transparency, color, bottom substrate).

Various automatic approaches have been suggested to reduce human error and cost-effort during manual image screening for detection and classification of litter from RGB aerial images. Deep learning is an approach that uses a large image dataset as training material to apply an algorithm into an unknown dataset. Studies testing deep learning for the detection of ML, both floating and underwater, require good quality images for training, testing, and validating the algorithms, and the lack thereof is a common constraint faced in these studies (Fulton et al., 2018; Garcia-Garin et al., 2021; Moorton et al., 2022). Thus, obtaining RGB data is highly important to provide these datasets and the understanding of how different water conditions (water color, transparency, depth), drone settings (flight height), and item characteristics (color and size) play a role becomes important for the further development of these image analyses techniques.

Kylili et al. (2019) developed an algorithm based on convolutional neural networks (CNN) to test the detection and classification of floating macrolitter (> 2.5 cm) on a test dataset of 2400 close-up images from various angles, reaching an accuracy of 86% and able to classify litter into 3 categories: plastic bottles, buckets, and straws. Similarly, Garcia-Garin et al. (2021) went a step further and tested a CNN algorithm to detect real floating litter at sea from RGB images obtained from aerial drones and aircraft at flight heights between 20 and 300 m, obtaining a detection accuracy > 81%. The authors made a user-friendly R-based version available and it is, to our knowledge, the first deep learning algorithm for floating ML freely open to the public. Nevertheless, litter classification is still weak in these studies and manual screening is therefore still a more reliable method for this purpose.

### Methodological improvements

The experiment of aerial detection of submerged items followed a different experimental design. Here, items were deployed in crosses instead of randomly in a frame. This potentially led to a learned bias during image analysis once evaluators saw the shape in which items were deployed. Although the other experiments followed a randomized position within the net/frame, the quantity of items per size was always the same which may have led to a similar learned bias during manual image screening of several images. In both cases, future research should use experimental designs with randomized position of items as well as randomized number of items per setting.

During our experiment only one shape was considered (square) and this was also 2-dimensional. The test of other shapes (lines/ropes, circles, non-uniform) and 3-dimensional items would be beneficial to derive results closer to reality. Similarly, the pictures in our experiments were taken with the camera perpendicular to the ground; testing a camera angle at 45° would give more information on the 3-dimensional shape of items floating on the water surface or on the seafloor, as well as increase area coverage per image.

Application of a light source pointing at items underwater can be subject to light scattering (i.e., light reflection) and requires that the camera be at a close distance to the item, since also light attenuation (i.e., loss of intensity) causes color change with distance (Bazeille et al., 2012; Bianco et al., 2015). Our detection analysis on items underwater based on color was limited to a distance in which the color tone was not changed. Studies suggest that item detection underwater should be based on an adapted color palette which considers these changes in tone (Bazeille et al., 2012) or correct these (Bianco et al., 2015; Mooney & Johnson, 2014) to improve item identification.

### Applicability of drones for official marine litter monitoring of coastal waters and its cost-efficiency

The MSFD requires that floating and seafloor litter be investigated with respect to “composition, spatial distribution and, where possible, sources,” defining priority areas, and improving photo or video tools for seafloor monitoring, considering monitoring protocols where size ranges, spatial, and temporal resolution are established as well as tested in different environmental conditions (JRC, 2013). Furthermore, there is a need to integrate data from in situ measurements, citizen science, and remote sensing, which could enable the implementation of the suggested Integrated Marine Debris Observing System (IMDOS) that shall couple models, satellites, and in situ observations for monitoring and assessment of the marine environmental status (Maximenko et al., 2019). Such system requires the need for in situ data for validation and calibration and also aims at testing new technologies to increase high-quality data and spatiotemporal resolution in regional monitoring (Maximenko et al., 2019).

Prior work demonstrated that drones could overcome weaknesses of current monitoring methods and fill the gaps from in-situ monitoring by providing data in larger spatial and temporal scales (Topouzelis et al., 2021). One main constraint of floating macrolitter monitoring with, e.g., on-ship observation is the short-visual observation time frame (JRC, 2013). With UAV data, this one can be extended and analysis can be repeated at a later stage. In terms of seafloor monitoring, e.g., scuba diver surveys are limited by high costs and effort, operative depth (< 50 m) and safe water conditions and do not give enough spatial distribution data (Madricardo et al., 2020). ROVs are more flexible at sites of high seafloor complexity and can gather additional information of the underwater environment, where hotspots as well as priority areas for long-term monitoring and management can be determined. Drone-based surveys are often regarded as highly cost-efficient in contrast to current methods; however, there is few studies estimating cost-efficiency in numbers. One study compared aircraft-based real-time observations versus RGB images obtained from aircrafts suggesting that the detection of ML items from manual screening of images involved lower effort (24 h versus 26–34 h) considering survey and analysis time (Garcia-Garin et al., 2020b), however without considering equipment costs and the implementation of a long-term monitoring. Furthermore, the images obtained from an aircraft have lower image resolution and focus on larger litter items, thus requiring shorter analysis time than drone-based surveys.

A summary of the parameters that can be covered by current methods versus UAVs and ROVs is illustrated in Table 4, based on literature and own experience. Similar to current methods, there are various constraints for drones, such as short battery life, permissions to operate, expensive equipment (when using multispectral sensors), longer initial implementation time, and higher expertise for image analysis, which call for a discussion on the efficiency between both methods. The assessment on cost-efficiency in our study is based on our own experience in the Baltic Sea, thus shall serve as an adaptable template for practitioners, subject to changes in staff costs per country, new technology advancements, improvements in the sampling protocol, equipment costs, etc. Our efficiency assessment is also highly subjective: evaluators tend to avoid using extreme values for evaluation (1: low to 3: high) and evaluators themselves may be biased and intent to portray one method better than another. Furthermore, the importance given to each efficiency criteria (accuracy, reproducibility, flexibility, and quality) and perception towards it can be based on context and vary between evaluators, as seen in the study by Andriolo et al. (2021).

**Table 4:** Data that can be gathered during floating and benthic marine litter with current marine litter monitoring methods (on-boat observation and scuba divers) versus aerial and underwater drone methods, based on JRC (2013), Buckland et al. (2001), Cheshire (2009), and own experience

**Parameters**	**On-boat observation**	**UAV (RGB)**	**Scuba divers**	**ROV (RGB)**
Observation time	Short	Can be repeated	Litter collected	Can be repeated
Can determine size ranges (e.g. 5 – 10 cm, 10 – 20 cm, etc.)?	Yes	Yes	Yes	Yes
Can determine item shape and color?	Yes	Yes	Yes	Yes
Can determine item type?	Limited	Limited	Yes	Yes
Can determine item material?	Limited	No	Yes	Limited
Can determine age/weathering?	Limited	No	Yes	Limited
Can estimate item density (number of items per unit area)?	Limited spatial coverage	Yes	Limited spatial coverage	Yes
Can estimate hotspots?	Limited spatial coverage	Yes	Limited spatial coverage	Yes
Can estimate sources?	Yes	Limited image resolution	Yes	Limited image resolution
Georeferencing	Limited	Yes	Limited	Limited
Equipment needed	Several	One	Several	One

Taking this into consideration, the assessment considered both UAV and ROV methods within the same cost-efficiency category as current methods (final score: moderate) for floating and seafloor monitoring. In this sense, current monitoring methods are sufficient to obtain data for monitoring; however, as more temporal and spatial data are needed, remote-sensing methods will be favored in the future. This will highly depend on improvements in the technology (e.g., battery capacity, sensors), availability of open access analysis techniques as well as adaptations of legal restrictions (permissions to operate). The application of remote-sensing and automatic recognition methods requires a longer initial set-up time: pre-processing of images, set up of image libraries, testing, validation, and calibration of algorithms, all requiring high expertise (Topouzelis et al., 2021). However, the running time is much lower in the long term. In contrast, manual screening methods require a short set-up time comprised of a short training session of volunteers with low experience; however, long-term analysis will be time-consuming. Further research should urgently aim at deriving user-friendly and open access methodologies for automatic recognition methods for the use of managers and officials. A centralized approach would be most useful to ensure harmonization and transferability.

A monitoring for coastal waters with drones could then follow the stratified sampling approach, as suggested for the open sea by Mace (2012). Here, coastal waters can be studied to determine the hotspots for plastics accumulation based on ocean dynamics, wind, geomorphology, scanned through models and satellite imagery (Leonard & Lucas, 2020). Later, UAVs can be used to proof the existence of these accumulation areas and together with ROVs assess litter abundance and composition through image acquisition and further analysis. For quality assurance and control, ground truth data of the floating and seafloor litter shall be taken through current methods which can also serve to get details on item identification and sources.

## Conclusion

Increasing ML inputs to coasts and seas demand for the improvement of monitoring technologies to acquire more spatial and temporal data on the amounts, composition, sources, and distribution of ML to derive management measures. Within the Marine Strategy Framework Directive, the identification of litter accumulation sites and priority areas, characterization of the most important sources of pollution and the further development of automatic methods for image and video analysis is relevant. In this study, we intended to provide an insight on the factors that influence the detection of ML in RGB images from aerial and underwater drones, considering water conditions (water transparency, color, depth, bottom substrate), item characteristics (color and size), and method settings (flight/dive height). Floating items > 5 cm length were detected with > 90% accuracy at flight heights of 20 m, with item color and water color playing an important role in detection. Submerged items > 10 cm were best detected (> 90%) at 20 m flight height, with water transparency, water depth, and water color influencing detection. Items on the seafloor were best detected (> 90%) at 1–2 m dive height and water transparency and type of bottom substrate were the main influencing factors for detection. Colors best detected were red in floating items and white and yellow in submerged items, whereas the detection of colors for items underwater varied largely upon water conditions (transparency, color, bottom substrate). Results showed that the application conditions for aerial and underwater drone methods do not overlap 100%, thus their complementary use needs to consider this. Future studies should consider items of 3-dimensional shape, the attenuation of colors underwater and larger sizes which could be detected from higher distance. The understanding of the influence of these factors in the detection of items in RGB images as well as for manual screening is highly relevant since they provide the database for automatic image analysis techniques, such as deep learning. As drone technologies continue to be tested and developed, these can work complementarily with standard methods to tackle weaknesses and improve cost-efficiency. A monitoring for coastal waters with drones could follow the stratified sampling approach, where oceanographic data, models and UAVs can be used to detect sites of litter accumulation and together with ROVs assess litter abundance and composition, which would be cross-validated by subsamples of floating and seafloor litter observed with current methods. Cost-efficiency suggests that both UAV and ROV methods lie within the same cost and efficiency category as current on-boat observation and scuba diving methods, and thus shall be considered for further testing in real scenarios for official marine litter monitoring methods.

## Supplementary Information

Below is the link to the electronic supplementary material.

Supplementary file1 (JPG 198 KB)

Supplementary file2 (JPG 203 KB)

Supplementary file3 (JPG 192 KB)

Supplementary file4 (JPG 212 KB)

Supplementary file5 (DOCX 12 KB)

## Data Availability

The original contributions presented in the study are included in the article as well as in the Supplementary Material.

## Footnotes

1 The Warnow estuary, during the period of 2007–2012, had total nitrogen annual content in the upper water layer (0–10 m) was 45.77 µmol l^−1^ (threshold value of 17.7 µmol l^−1^), total phosphorus was 1.56 µmol l^−1^ (threshold value 0.59 µmol l^−1^) and chloropyll-a had a concentration of 8.3 µg l^−1^ in summers of 2011–2016 (threshold value 2.4 µg chl-a l^−1^). Coastal waters between Warnemünde and Darß had a total nitrogen of 20.32 µmol l^−1^ (threshold value 19.5 µmol l^−1^), total phosphorus of 0.86 µmol l^−1^ (threshold value 0.65 µmol l^−1^), and chlorophyll-a concentration of 1.91 µg chl-a l^−1^ (HELCOM, 2018a, 2018b, 2018c).
