# Supplementary figures and images for: Aerial and underwater drones for marine litter monitoring in shallow coastal waters: factors influencing item detection and cost-efficiency
Source: Environ Monit Assess. 2022 Oct 11;194(12):863. doi: 10.1007/s10661-022-10519-5 (PMC9553762; doi:10.1007/s10661-022-10519-5)

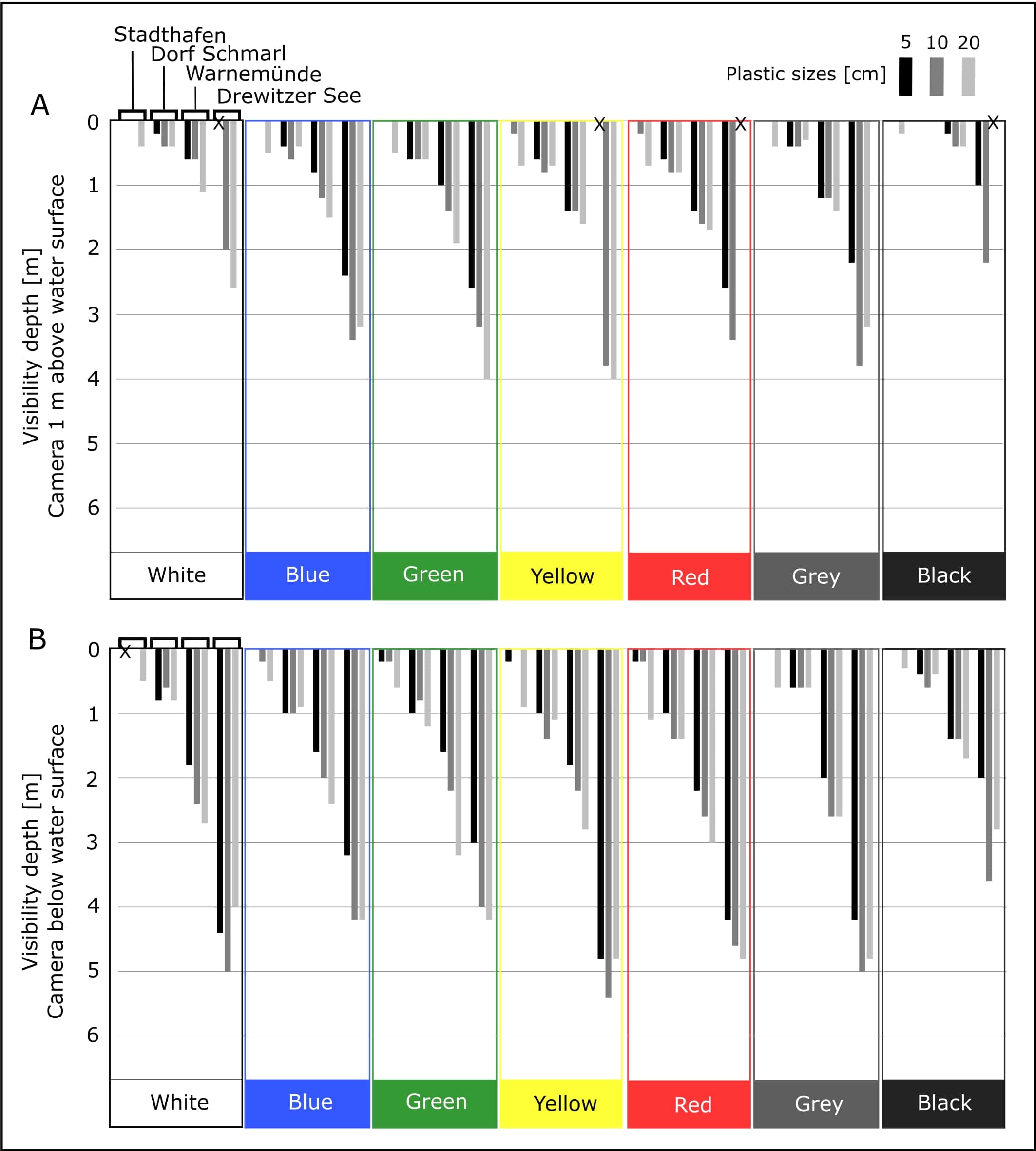

Supplement: Supplementary file 1 — Supplementary file1 (JPG 198 KB) [file 10661_2022_10519_MOESM1_ESM.jpg]

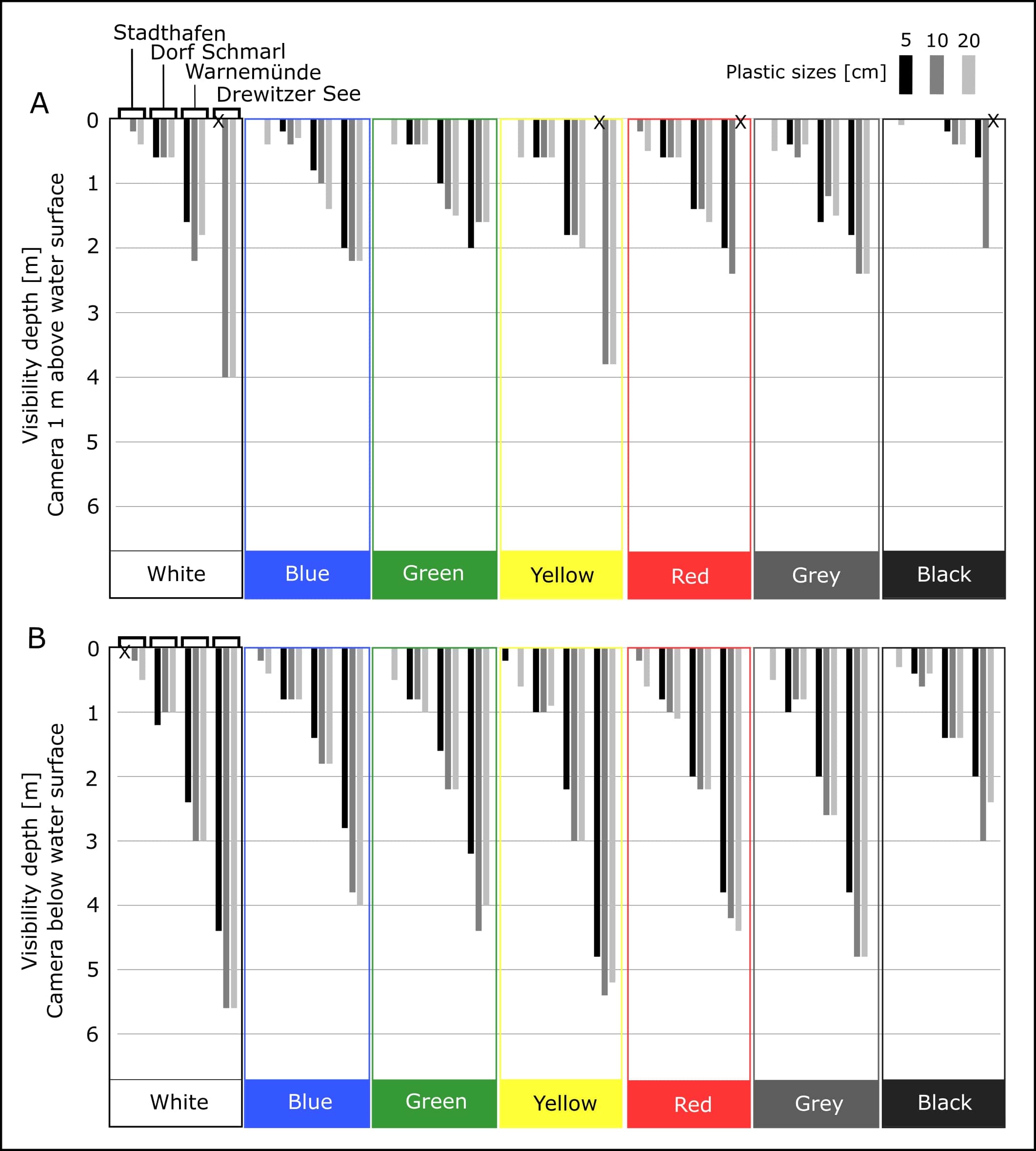

Supplement: Supplementary file 2 — Supplementary file2 (JPG 203 KB) [file 10661_2022_10519_MOESM2_ESM.jpg]

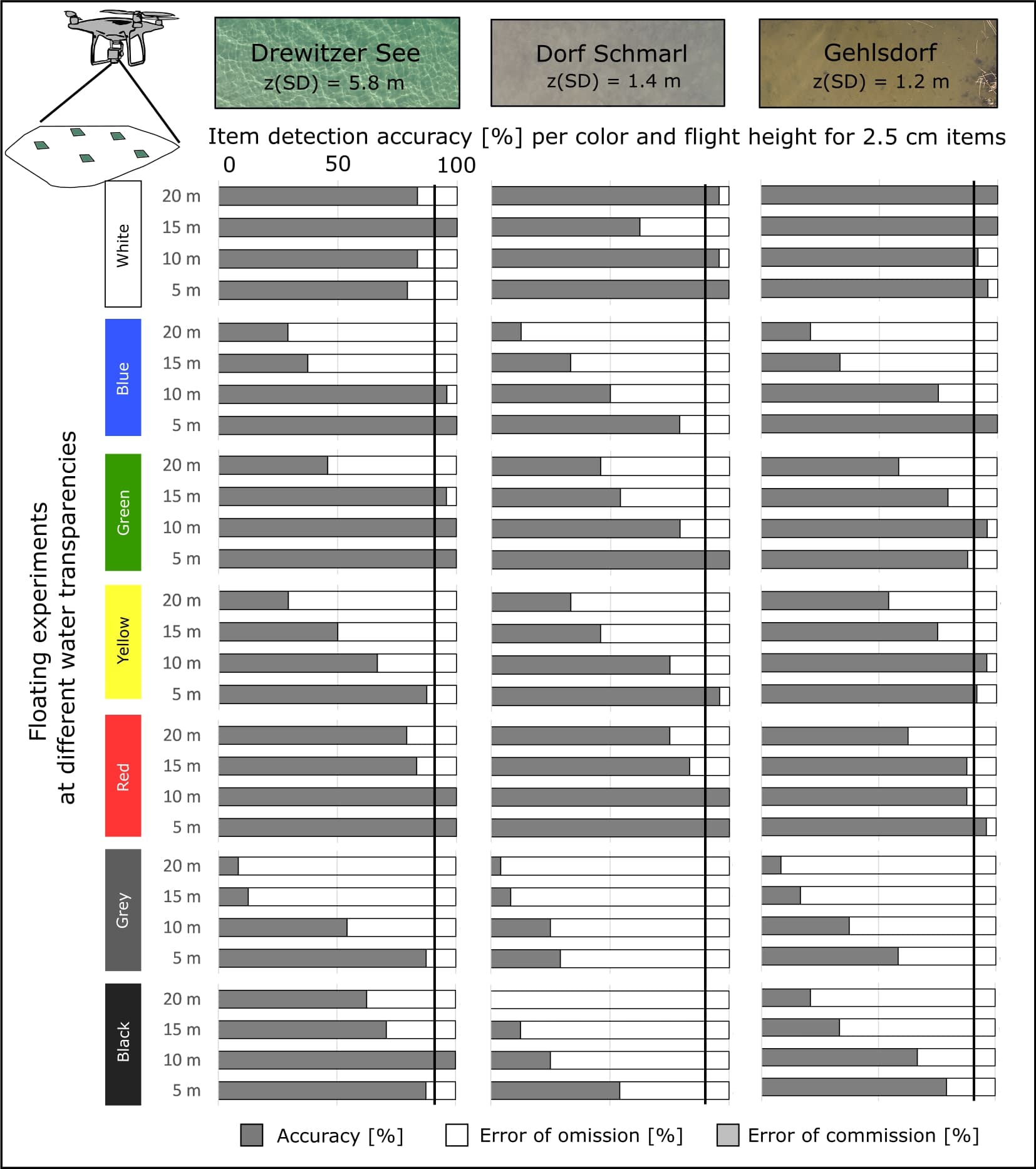

Supplement: Supplementary file 3 — Supplementary file3 (JPG 192 KB) [file 10661_2022_10519_MOESM3_ESM.jpg]

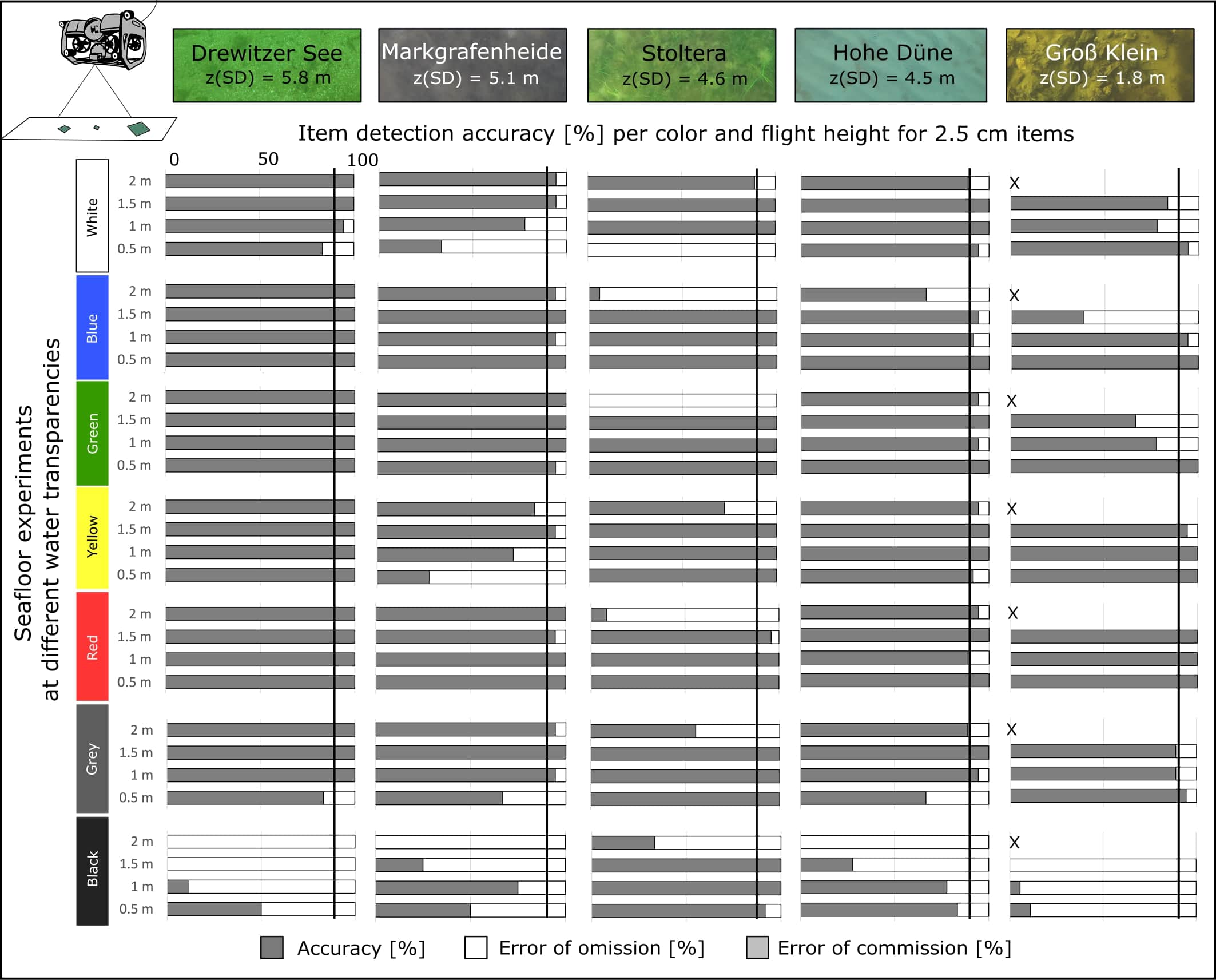

Supplement: Supplementary file 4 — Supplementary file4 (JPG 212 KB) [file 10661_2022_10519_MOESM4_ESM.jpg]
