# Supplementary material for: Aerial and underwater drones for marine litter monitoring in shallow coastal waters: factors influencing item detection and cost-efficiency
Source: Environ Monit Assess. 2022 Oct 11;194(12):863. doi: 10.1007/s10661-022-10519-5 (PMC9553762; doi:10.1007/s10661-022-10519-5)
Supplement: Supplementary file 5 — Supplementary file5 (DOCX 12 KB) [file 10661_2022_10519_MOESM5_ESM.docx]

**Figure S1**: Median maximum visibility depth [m] for items of different sizes (5, 10 and 20 cm) and 7 colors. The assessment considered the maximum depth at which the original colors were visible without change in tone. The experiment was carried out with the camera held at (A) 1 m above water surface and (B) right below water surface at sites of different water transparency: Stadthafen (polytrophic), Dorf Schmarl (eutrophic), Warnemünde (mesotrophic) and Drewitzer See (oligotrophic) (Fig. 1).

**Figure S2.** Median maximum visibility depth [m] for items of different sizes (5, 10 and 20 cm) and 7 colors. The assessment considered the maximum depth at which the square shape was clearly visible. The experiment was carried out with the camera held at (A) 1 m above water surface and (B) right below water surface at sites of different water transparency: Stadthafen (polytrophic), Dorf Schmarl (eutrophic), Warnemünde (mesotrophic) and Drewitzer See (oligotrophic) (Fig. 1).

**Figure S3.** Accuracy and error of detection [%] for each color per flight height for the floating recovery experiments at sites of different water transparency (Fig. 1), for items of 2.5 cm presenting all colors in one image.

**Figure S4.** Accuracy and error of detection [%] for each color per dive height for the underwater recovery experiments at sites of different water transparency (Fig. 1), for items of 2.5 cm presenting all colors in one image.
